# Considerations on the Rational Design of Covalently Conjugated Cell-Penetrating Peptides (CPPs) for Intracellular Delivery of Proteins: A Guide to CPP Selection Using Glucarpidase as the Model Cargo Molecule

**DOI:** 10.3390/molecules24234318

**Published:** 2019-11-26

**Authors:** Yasaman Behzadipour, Shiva Hemmati

**Affiliations:** 1 Department of Pharmaceutical Biotechnology, School of Pharmacy, Shiraz University of Medical Sciences, Shiraz 71345-1583, Iran; yasaman_behzadipour@yahoo.com; 2 Biotechnology Research Center, Shiraz University of Medical Sciences, Shiraz 71345-1583, Iran; 3 Pharmaceutical Sciences Research Center, Shiraz University of Medical Sciences, Shiraz 71345-1583, Iran

**Keywords:** cell-penetrating peptides, computational drug design, peptide-based drug delivery, therapeutic peptide-protein, bioconjugation, biomacromolecules, protein delivery, carboxypeptidase G2

## Abstract

Access of proteins to their intracellular targets is limited by a hydrophobic barrier called the cellular membrane. Conjugation with cell-penetrating peptides (CPPs) has been shown to improve protein transduction into the cells. This conjugation can be either covalent or non-covalent, each with its unique pros and cons. The CPP-protein covalent conjugation may result in undesirable structural and functional alterations in the target protein. Therefore, we propose a systematic approach to evaluate different CPPs for covalent conjugations. This guide is presented using the carboxypeptidase G2 (CPG2) enzyme as the target protein. Seventy CPPs —out of 1155— with the highest probability of uptake efficiency were selected. These peptides were then conjugated to the *N*- or C-terminus of CPG2. Translational efficacy of the conjugates, robustness and thermodynamic properties of the chimera, aggregation possibility, folding rate, backbone flexibility, and aspects of in vivo administration such as protease susceptibility were predicted. The effect of the position of conjugation was evaluated using unpaired t-test (*p* < 0.05). It was concluded that N-terminal conjugation resulted in higher quality constructs. Seventeen CPP-CPG2/CPG2-CPP constructs were identified as the most promising. Based on this study, the bioinformatics workflow that is presented may be universally applied to any CPP-protein conjugate design.

## 1. Introduction

Biotherapeutics such as protein-based therapeutics are a fast-growing group of pharmaceuticals [1]. Although the advantage of biotherapeutics is their target specificity, one of the major challenges in the way of further development of protein-based therapeutics is their intracellular delivery. A huge hydrophilic proteinaceous macromolecule cannot cross the hydrophobic lipid bilayer membrane surrounding a cell [2]. This obstacle, however, can be overcome with the assistance of a cell-penetrating peptide (CPP).

CPPs are short relatively non-toxic peptide sequences, usually less than 30 amino acids, which not only have the ability to cross cellular membranes, but also can co-transport a variety of biologically active molecules (cargoes) inside the cells [1,2]. Since their discovery in 1988, a wide range of different CPPs has been identified and subjected to clinical trial studies [3,4]. Chemically, CPPs are categorized into three classes, known as cationic, hydrophobic, and amphipathic. Cationic CPPs have positively charged residues that are capable of interacting with negatively charged groups on the cellular membrane, which results in their internalization [5]. Hydrophobic CPPs have mainly nonpolar residues that can interact with the hydrophobic segments of the membrane [6]. Despite the presence of both polar and non-polar residues in amphipathic CPPs, some non-amphipathic peptides can display amphipathic behavior after folding into α-helices or β-sheets [7].

Considering CPPs as appropriate delivery vectors for proteinaceous therapeutics [8], two main strategies are available to enable CPPs for the co-transportation of the desired cargo molecules across the plasma membrane. These systems might be designed in the form of covalent conjugation of the CPP and the cargo molecule or as a non-covalent complex of CPP and cargo [9]. Covalent conjugation can be established using chemical synthesis methods [10,11,12]. However, for protein or peptide cargos, covalent conjugation to CPPs can be accomplished by expressing the chimeric gene in bacterial expression systems such as *E. coli* [13,14].

There are some other limitations and challenges regarding protein-based therapeutics, such as low solubility, short half-life, and the induction of unwanted or fatal allergic and immunological responses in the recipients. For engineered CPP-protein conjugates, wherein new amino acid orders are added to the target protein, some further issues in addition to the above-mentioned challenges must be addressed, including incorrect folding of the engineered protein, changes in 3D structures and backbones, and variations in the side chains that stabilize the structure [15,16].

Although more than one thousand experimentally validated CPPs have been identified [17], a universal method to discriminate between CPPs and designate the ideal peptide for a conjugation is not currently available. Arbitrarily selecting a frequently used CPP might not necessarily result in the design of an optimal bio-conjugate [18]. Therefore, in the covalent conjugation of CPPs to a protein, there are several issues that still need to be addressed in the process of CPP selection, including: (1) consideration of CPPs that provide a higher penetration of the conjugate; (2) evaluating the degree of CPP interference with the stereochemistry of the conjugate; (3) pondering CPPs with higher solubility and stability in the production and formulation process; (4) assessing CPPs that do not result in the aggregation of the cargo after delivery; and (5) inspecting the degree of potential allergenicity, protease sensitivity, and hemolysis of the conjugate. Hence, devising a realistic workflow to follow the aforementioned criteria in the selection process of the appropriate CPP is one of the most influential factors affecting the structural and functional properties of the target protein.

Using a guideline considering computational approaches in the rational design of a chimera would provide the opportunity to envisage part of the abovementioned criteria and attain a comprehensible understanding of the protein’s characteristics [19,20]. Herein, the credit goes to the improvement in the software or web-based portals with strong algorithms that determine the structure of the conjugates and predict the in vivo effects of the chimera.

Therefore, we propose a bioinformatics guideline using some already existing in silico methods to select the best CPP candidates in conjugation with carboxypeptidase G2 (CPG2) enzyme as the model cargo molecule. CPG2 also known as glucarpidase (Voraxaz^®^) is an FDA-approved bacterial enzyme breaking methotrexate (MTX) down into two noncytotoxic metabolites [21,22,23]. Due to its capacity for hydrolyzing a wide range of folate analogs, CPG2 is also able to convert non-toxic glutamated nitrogen mustard prodrugs into cytotoxic substances; hence, it is proposed as an intriguing choice for the antibody/gene-directed enzyme prodrug therapy modalities known as ADEPT and GDEPT, respectively [24,25,26]. In glucarpidase therapy, the enzyme only reduces the circulating MTX levels and has no access to the drug inside the cells. Therefore, there is a rebound of MTX into the blood due to the release of intracellular stored MTX [27]. Accordingly, it seems reasonable to deliver the CPG2 enzyme to the intracellular compartment. Through experimental studies, we have recently achieved the intracellular delivery of CPG2 in conjugation with the well-known transcription transactivator protein of HIV-1 (TAT) peptide [28]. Therefore, CPG2 has been used as the model cargo in the preparation of the current investigation.

Within this study, we describe significant factors affecting CPP-CPG2 recombinant conjugate and will introduce a guideline for the design of CPP-protein conjugates. This instruction would provide a workflow using available bioinformatics tools to design an effective biotherapeutic with the optimum pharmacological responses and the fewest side effects. It is expected that this guideline will be useful for scientists of different disciplines regarding protein engineering and delivery.

## 2. Results and Discussions

### 2.1. Primary Dataset and Penetration Prediction of CPPs

Unique linear CPPs with natural L-conformation amino acids were retrieved from CPPsite 2.0. In general, CPPs with the highest uptake efficiency are preferred for conjugation. Hence, CPP sequences were submitted to the CPPred-RF server to determine the degree of cellular uptake (Table S1). The CPPred-RF webserver is a two-layered prediction engine. The first layer is based on some features, such as physicochemical properties and dipeptide composition. This layer defines if the submitted sequence is cell-penetrating. When the peptide is cell-penetrating, then the second layer of prediction estimates the uptake of the peptide by cells as high or low. In each layer, prediction confidence is reported. Finally, 70 peptides that were predicted to have the highest uptake efficiency with the prediction confidence of above 0.9 were subjected to further analyses (Table 1). We have checked whether the 70 top selected CPPs have shown adequate uptake efficiency in experimental studies. The data on previously studied top CPPs, as well as the category of each CPP, are available in Table 2. Levels of uptake for 66 out of 70 CPPs were available from laboratory experiments. As presented in Table 2, all of the 70 selected CPPs have displayed either high or medium uptake efficiency.

**Figure 1:**
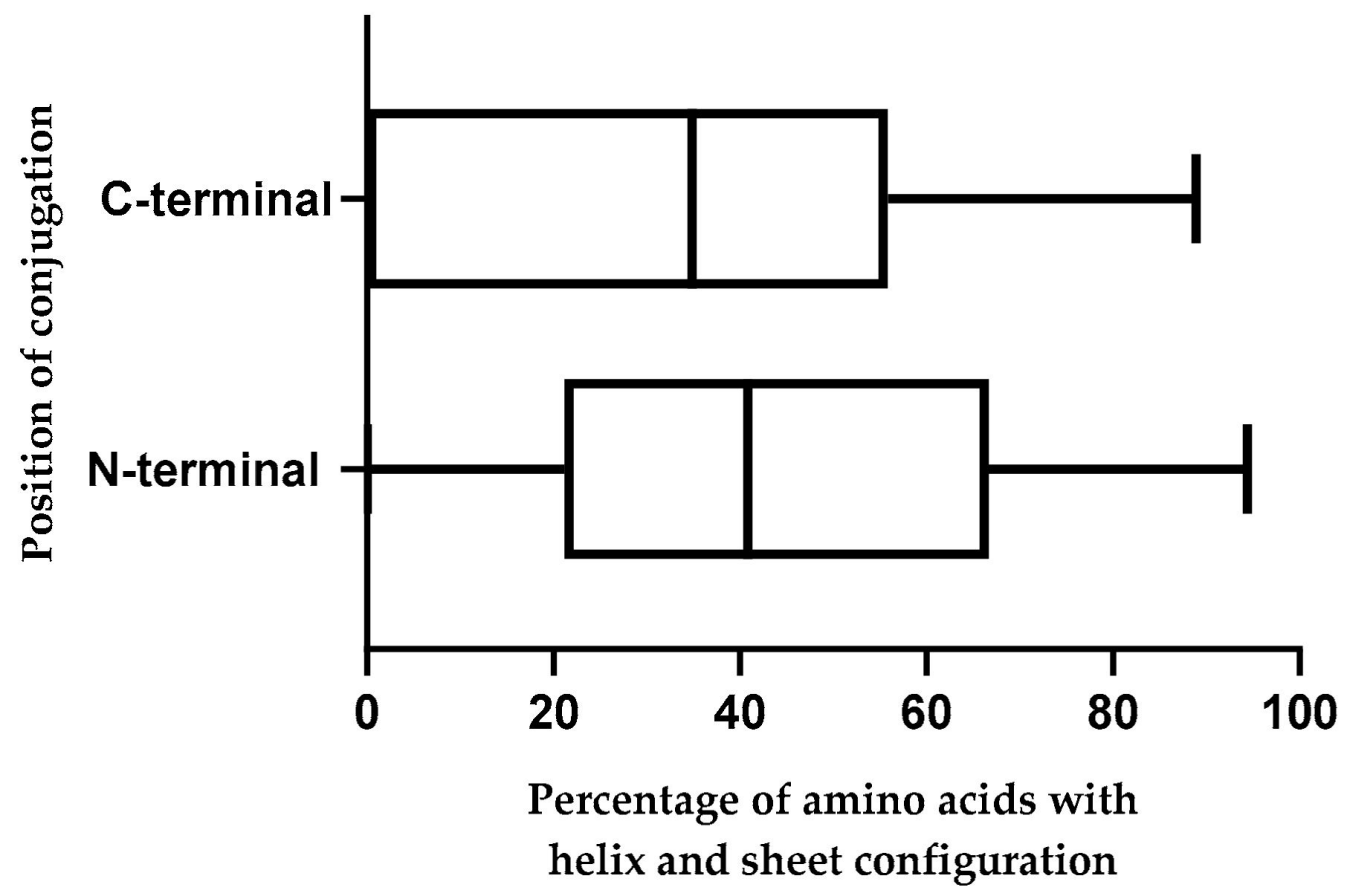
The percentage of distribution of amino acids with helix and sheet configuration in the N-terminal (CPP-CPG2) and C-terminal (CPG2-CPP) conjugates.

There is some evidence available that the secondary structure of an amphipathic CPP is correlated to its uptake efficiency [46,47,48]. Therefore, secondary structures of the top 70 CPPs after conjugation with CPG2, for both N- and C-terminal positions were recorded. When a CPP folds into an α-helix or β-sheet it potentially penetrates the cells more efficiently. Therefore, the percentage of amino acids that are able to form helices and sheets was calculated for the CPP fragment within each conjugate (Table 3). According to the acquired results, peptides 3, 4, 6, 14, 56, and 62 in N-terminal and peptides 2, 14, 43, 52, 56, and 58 in C-terminal conjugation have more than 85% amino acids with helix and sheet configuration. It should be noted that compared with the C-terminal conjugation of CPPs, N-terminal homologs resulted in a significantly higher percentage of amino acids with the helix and sheet configuration (Figure 1).

### 2.2. mRNA Secondary Structure Prediction of cpp-cpg2/cpg2-cpp Conjugates

**Figure 2:**
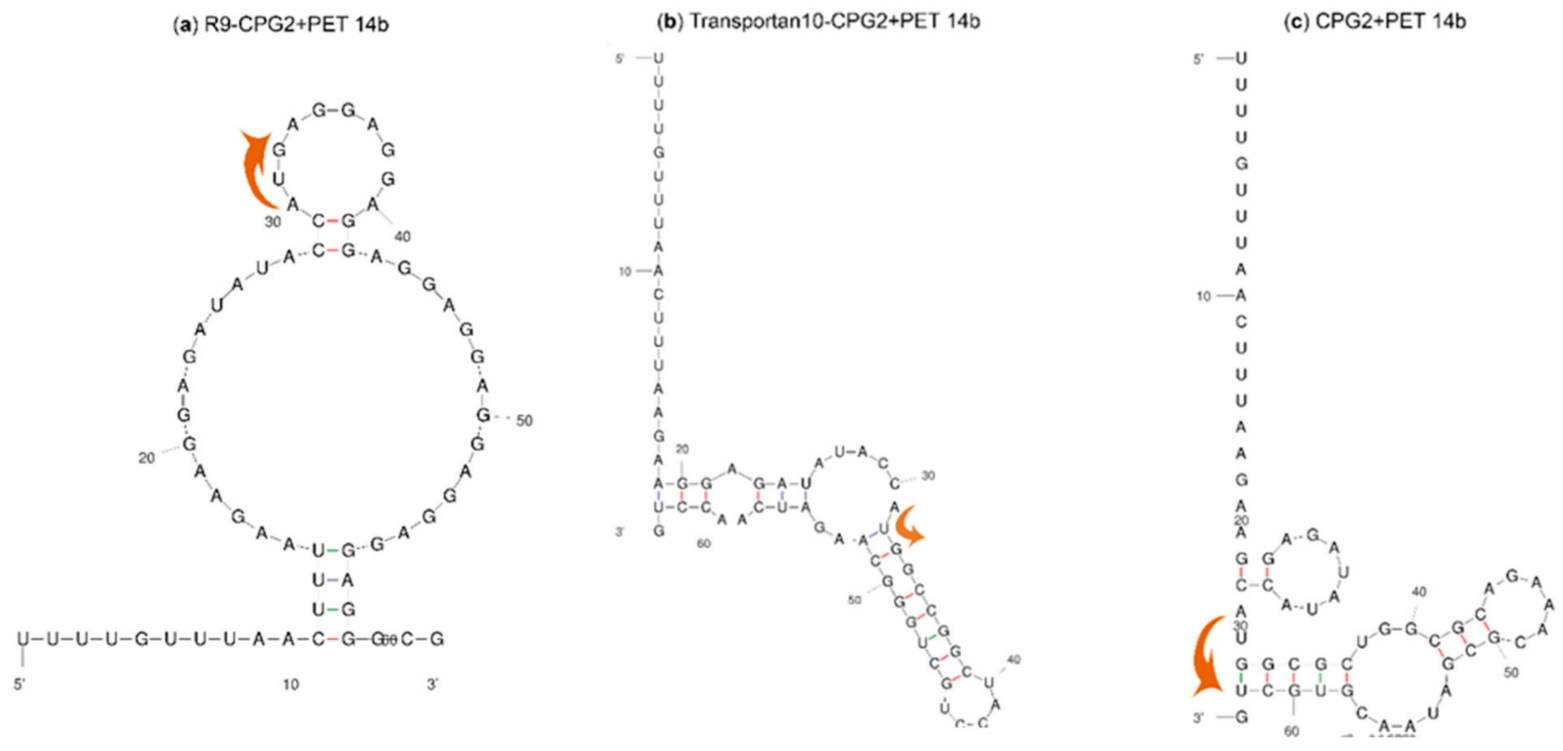
mRNA initiation translation region secondary structures and location of the start codon (indicated by arrows) for two conjugates and CPG2. (**a**) “R9-CPG2 + PET 14b” has a higher *ΔG* value compared with CPG2 and the start codon is exposed (**b**) “Transportan 10-CPG2 + PET 14b” has a lower *ΔG* value compared with CPG2 and the start codon is not exposed. (**c**) Control CPG2 + PET 14b: start codon is not exposed.

The stability of the mRNA structure affects the expression level of proteins. A more positive *ΔG* value of the mRNA secondary structure in the translation initiation region correlates with a higher probability of the translation of mRNA into protein [49]. The obtained *ΔG* values from the mfold server indicated that 74% of the N-terminal conjugates had higher *ΔG* values compared with the unconjugated CPG2. N-terminal conjugates from peptides 10, 18, 31, 36, and 61 displayed the highest *ΔG* values. Besides *ΔG*, the level of exposure of the AUG start codon in the mRNA secondary structure has a regulatory effect on the rate of protein translation in *E. coli*. If the start codon locates on a loop, higher exposure to the ribosomal subunit occurs and results in a higher level of translation compared with a start codon on the stem [50]. In this study, in 50% of the N-terminal conjugates, the start codon was located on a loop and in the rest of the conjugates, as well as CPG2 itself, the starting codon was on the stem. Secondary structures of two conjugates and CPG2 are shown in Figure 2. The *N*-terminal conjugate of “R9-CPG2” has a high *ΔG* value (−1.6 kcal.mol^−1^) and an exposed start codon, while “Transportan 10-CPG2” *N*-terminal conjugate displays a low *ΔG* value (−13.2 kcal.mol^−1^) and its start codon is not exposed. *ΔG* values and position of the start codon for all of the conjugates are available in Table S2. It should be noted that all the seventy C-terminal conjugates have the same initiation translation region as the control unconjugated CPG2.

### 2.3. Physiochemical Properties of CPPs and CPP-CPG2/CPG2-CPP Conjugates

Various physiochemical properties were calculated for CPG2 and the top 70 CPPs both alone (Table S3) and in conjugation to CPG2 at the *N*-terminal (Table 4) and C-terminal (Table S4) positions via ProtParam tool. Top 70 CPPs’ lengths were between 5-22 amino acids. All the top 70 CPPs except number 69 have basic pI (Table S3), indicating an overall positive charge in the blood pH (7.4). The pI of unconjugated CPG2 was calculated to be 6.22. Only fusion proteins resulting from peptides 12, 66, 67, 68, and 69 had an acidic pI due to the anionic nature of CPPs (Table 4). The rest of the conjugates would have an overall positive charge at pH = 7.4. None of the conjugates had a pI near 7.4, indicating a low risk of protein aggregation due to pI after administration.

The instability index for all the conjugates is less than 40, indicating that the conjugates are probably stable in the test tube. However, only conjugates resulting from peptides 1, 3, 12, 14, 55, and 67 do not have elevated instability index compared with the unconjugated CPG2. GRAVY value for all the conjugates is negative, which means all of them are hydrophilic. Compared with CPG2, the GRAVY for conjugates resulting from peptides 1, 12, 14, 28, 29, 30, 32, 33, 66, 67, 68, and 69 showed higher values which could result in higher hydrophobicity. This might, in turn, leads to an increased chance of aggregation. All the conjugates composed of “CPP5s” and “pVEC mutants” showed an increase in GRAVY value compared with CPG2. There was no significant difference between the physiochemical properties of the N- and C-terminal conjugates.

### 2.4. The Solubility of CPP-CPG2 and CPG2-CPP Conjugates

The solubility of the top 70 CPPs conjugated to CPG2, regarding both N- and C-terminal conjugations were predicted using the ccSOL server (Table 5). The solubility of proteins is a crucial factor for the production, formulation, and delivery of protein-based therapeutics. Solubility is influenced by extrinsic and intrinsic factors. By optimizing extrinsic factors such as pH, ionic strength, the temperature of the solvent, and in the presence of various additives, protein solubility can be increased. The intrinsic factors are mostly related to the amino acids on the proteins’ surface [51]. CPPs have been used to increase the aqueous solubility of their cargo. For instance, the conjugation of taxol to CPPs resulted in the improvement of taxol’s poor solubility [52,53]. CcSol predicts the percentage of protein solubility. On average, conjugates from “CCP5s” and some “Crot derivatives” displayed an overall higher solubility score. Higher solubility of the “CCP5” family might be due to the increased negative charge on the protein’s surface associated with the anionic nature of these CPPs [51]. However, due to differences between results from different solubility prediction servers, further experimental validations are required to reach a definite conclusion. The *N*- and C-terminal CPP-CPG2 and CPG2-CPP conjugates had an average solubility of 79.69% and 79.44%, respectively. Therefore, the position of a conjugation had no significant effect on the solubility.

### 2.5. Three-Dimensional Modeling of CPP-CPG2 and CPG2-CPP Conjugates

CPPs might influence the structure and function of the covalently conjugated protein [54]. To deliver CPG2, the CPP segment in the conjugate should be exposed to interact with the functional groups on the plasma membrane. The CPP segment should not have any interactions with the active site of the enzyme to keep the functionality of the target protein unchanged. The conjugate should still have reasonable stereo-chemical characteristicw and low steric clashes leading to an easy folding. The I-TASSER program was used to generate PDB models. For each conjugate, models with the highest C-score value were selected and further analyzed by Ramachandran plots. In all models, the CPP domains were exposed and there were no interactions between CPPs and the active site of the CPG2 enzyme. 

**Figure 3:**
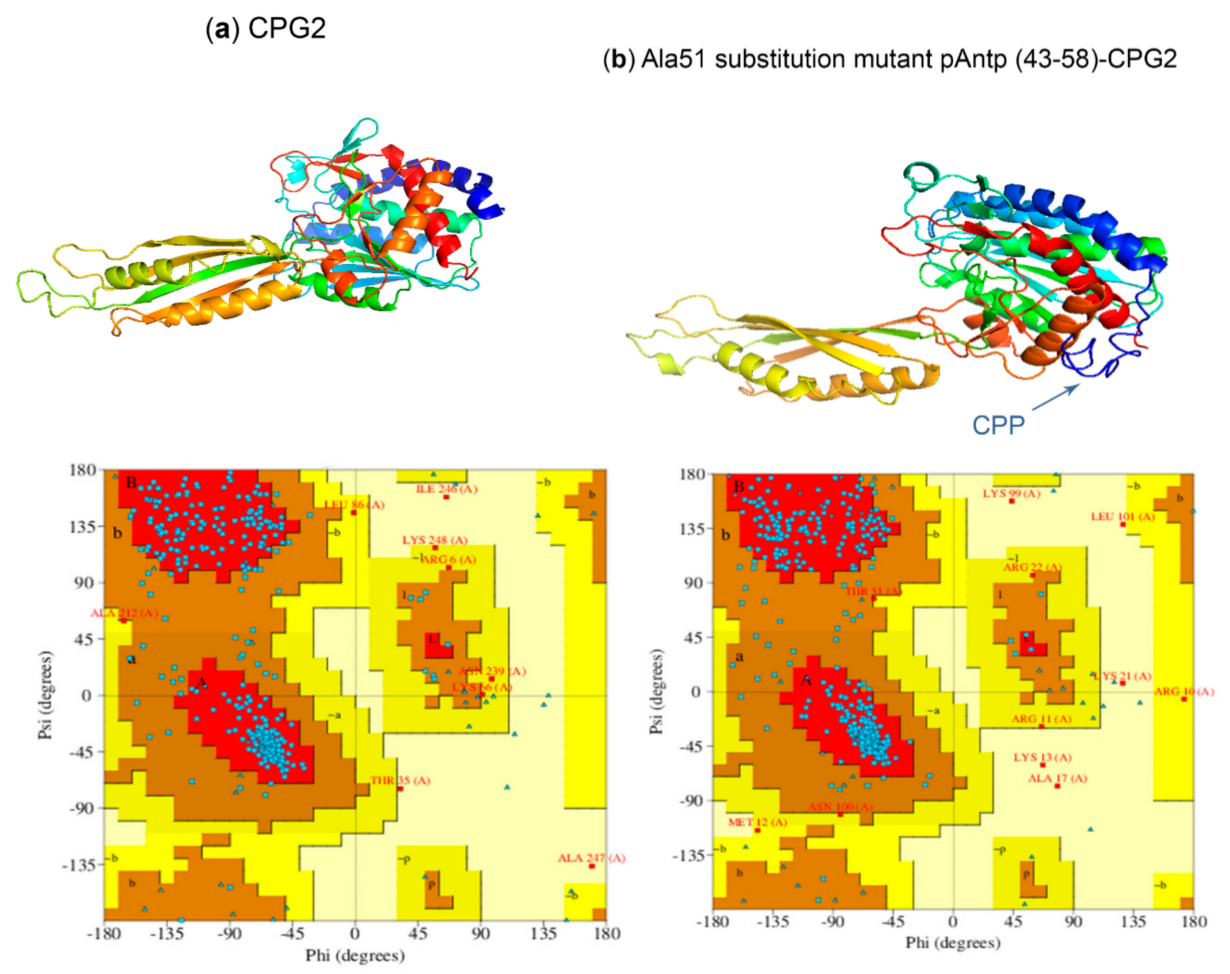
3D models of (**a**) CPG2 and (**b**) “Ala51 substitution mutant of pAntp (43-58)” conjugated to the *N*-terminal position of CPG2 and their respective Ramachandran plots.

**Figure 4:**
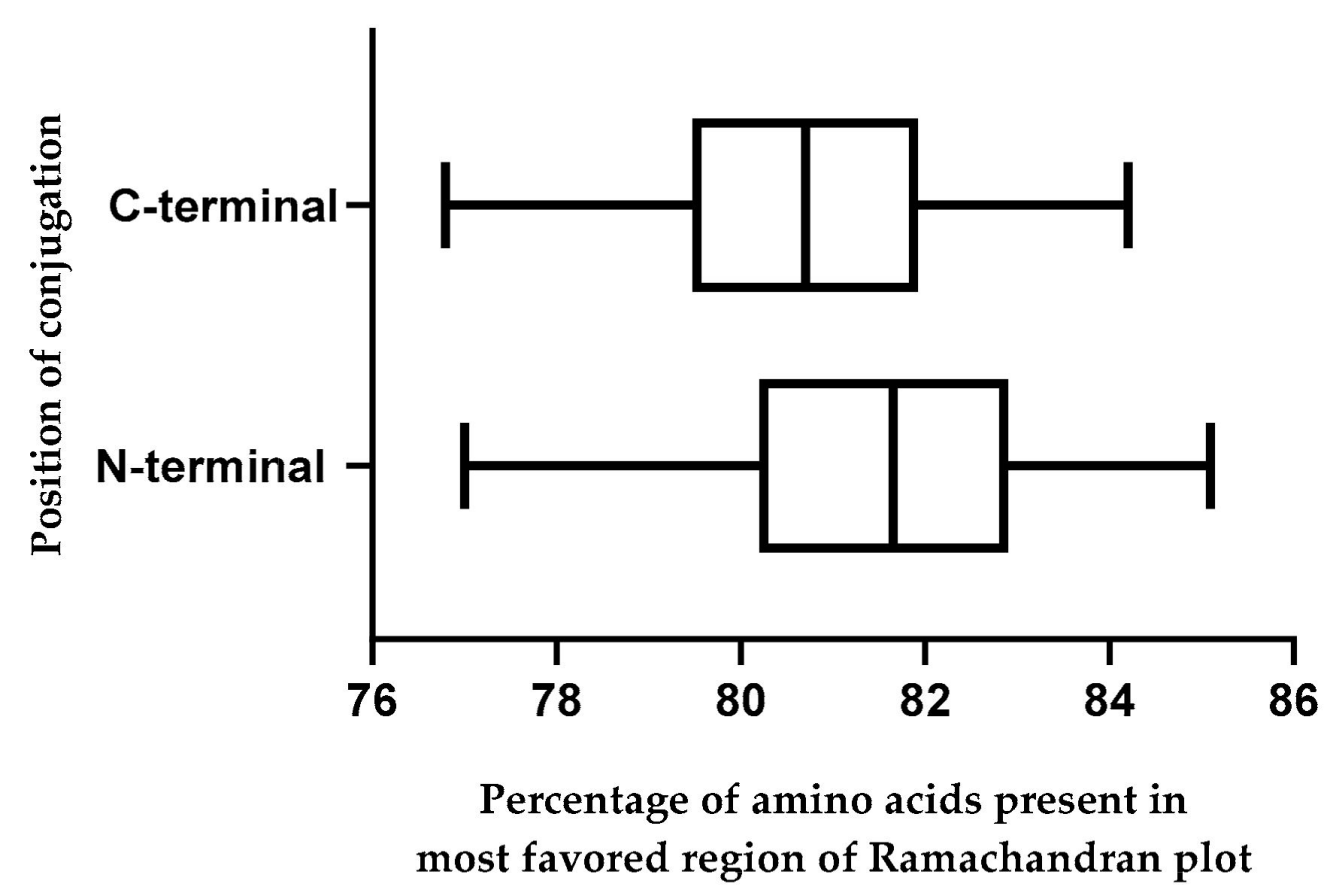
Distribution of percentage of amino acids present in the most favored region of Ramachandran plot across N- (CPP-CPG2) and C-terminal (CPG2-CPP) conjugates.

Residues in the most favored region and additionally allowed region of all Ramachandran plots exceeded 90%, indicating that all models were reliable [50,55,56]. Compared to CPG2, *N*-terminal conjugates from peptides 9, 16, 17, 25, 54, and 62 and C-terminal conjugates from peptides 29, 39, and 69 had a higher number of residues in the most favored region of their Ramachandran plots (Table S5). Therefore, it seems that the *N*-terminal conjugates of “pAntp (49–58)-CPG2”, “Ala51 substitution mutant of pAntp (43–58) -CPG2”, “Crot (27–39) derivative 7-CPG2”, “Crot (27–39) derivative 8-CPG2”, “Crot (27–39) derivative 10-CPG2”, and “Crot (27–39) derivative 14-CPG2”, as well as “CPG2-pVEC mutant 2”, “CPG2- Ala47 substitution mutant of pAntp (43–58)”, and “CPG2-Bip16” C-terminal conjugates, have easier folding than unconjugated CPG2 (Figure 3 and Table S5). Furthermore, it should be noted that *N*-terminal CPP-CPG2 conjugates had a significantly higher number of residues in the most favored region compared with the C-terminal isoforms (Figure 4). Accordingly, *N*-terminal conjugates have sterically higher robustness.

### 2.6. Thermodynamic Characteristics of CPP-CPG2 and CPG2-CPP Conjugates

Thermodynamic properties play an important role in developing stable biotherapeutics [57]. SCOOP server calculates thermodynamic quantities associated with the folding transition from unfolded to the native state, based on a protein’s 3D structure and the host organism (Table 6, Table S6). The calculated parameters are co-related via the Gibbs-Helmholtz equation. According to the report of Pucci et al. [58], three main strategies result in a more thermodynamically stable protein. A more negative enthalpy change (*ΔH_S_*) measured at the maximum stability temperature (*T_S_*) results in an overall decrease of *ΔG* at all temperatures. In the second strategy, the heat capacity upon folding (*ΔC_p_*) becomes less negative, which yields an increase in melting temperature (*T_m_*). The last strategy consists of an increase in *T_s_* defined at the minimum of the *ΔG(T)* curve. As a result, one can find the most stable conjugated protein at room temperature by comparing folding free energy values at room temperature (*ΔG_r_*). The conjugate’s stability at higher temperatures can be associated with their corresponding *T_m_*. Based on calculated values conjugates which have higher (or even equal) *ΔG_r_* and *T_m_* compared with CPG2 are resulted from N-terminal conjugates with peptides 16, 21, 22, 36, 44, and 45 and C-terminal conjugation with peptides 16 and 63 (Table 6 and Table S6). The position of conjugation does not significantly affect any of the thermodynamic values calculated by SCOOP.

The stability of the conjugates was also evaluated using the FoldX Suite server (Table 7 and Table S7). According to Rahmatabadi et al. [55], among the dynamic quantities calculated by FoldX Suite, four energies, including total free energy, side-chain hydrogen bonds, solvation polar, and van der Waals clashes have significant correlation with the number of amino acids in the most favored region of Ramachandran Plot; hence, affecting the stability of a protein. Amidst CPP-CPG2 conjugates, *N*-terminal conjugates derived from peptides 1, 19, 20, 21, 37, 64 and C-terminal conjugates composed of peptides 9, 32, 44, 47, 55, 56, 61, and 69 have the most negative *ΔG* values of side H bond energies. Only the *N*-terminal conjugate of “Bip 6-CPG2” has more thermodynamically stable energies across all 4 categories compared with the unconjugated CPG2. Furthermore, it was concluded that the position of conjugation does not significantly affect the calculated thermodynamic energies of CPP and CPG2 conjugates.

### 2.7. Prediction of the Aggregation Possibility of CPP-CPG2 and CPG2-CPP Conjugates

It has been demonstrated that depending on the surrounding conditions and structure, proteins can form insoluble, though stable constructs composed of amyloid fibrils or amorphous aggregates [59]. Protein aggregation is one of the troubles encountered in vitro and in vivo. Protein release and activity are distorted if aggregation occurs after delivery [60,61]. As a result, several computational strategies are used to determine the propensity of proteins to form amyloids based on their amino acid sequence [62,63,64]. Herein, we have employed two servers called Aggrescan and PASTA 2.0 to investigate if the addition of a CPP sequence to CPG2 affects the probability of protein aggregation. Aggrescan finds the number of hotspots for aggregation in a protein regarding amino acid composition, while PASTA 2.0 evaluates the chance of amyloid formations considering pairwise interactions within β-sheets. According to PASTA 2.0 calculations, there was no difference between the numbers of amyloid regions in the conjugates compared with unconjugated CPG2. However, based on Aggrescan analyses, 19 *N*-terminal conjugates and 15 C-terminal conjugates displayed 17 aggregation hotspots, whereas CPG2 itself and the rest of the conjugates had 16 aggregation hot spots. As a result, 19 *N*-terminal and 15 C-terminal conjugates might have an increased risk of aggregation than the non-fused CPG2 (Table S8).

### 2.8. Folding Rate and Backbone Flexibility of CPP-CPG2 and CPG2-CPP Conjugates

**Figure 5:**
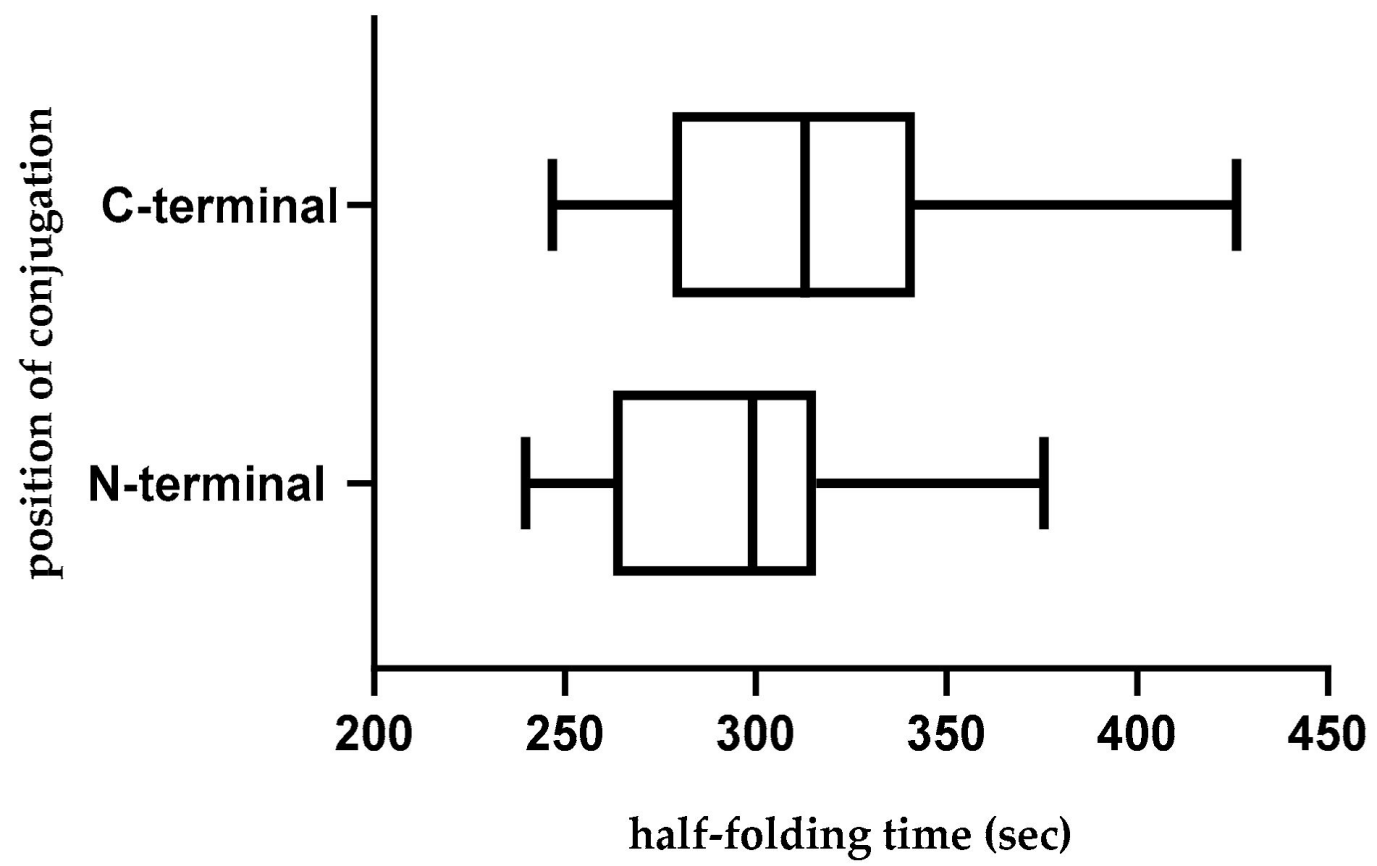
Distribution of half-folding time across *N*-terminal (CPP-CPG2) and C-terminal (CPG2-CPP) conjugates.

A protein chain has to be folded into its native conformation to be functional. Therefore, the conjugates must have folding rates closer to the unconjugated CPG2. This means that the attached CPP sequence should cause a minimum disturbance in the folding of a chimera. Although conjugates showed longer folding half-times compared with CPG2, *N*-terminal conjugates from peptides 8, 10, 18, 34, 36, 59, 66, 68, and 69 and C-terminal conjugates from peptides 12, 59, 60, 61, and 64 displayed the closest half folding time to the CPG2 itself (Table 8). Furthermore, regarding calculated values, *N*-terminal CPP-CPG2 conjugates displayed significantly lower half-folding time compared with C-terminal conjugates (Figure 5).

Proteins are composed of a string of amino acids, which after some non-covalent interactions fold into naturally flexible tertiary structures. The degree of flexibility is associated with a protein’s function and is crucial in protein engineering and rational drug design [65]. Ligand-binding sites in enzymes usually have both flexible and rigid residues. Rigid residues are associated with specificity and tightness of ligand binding, while flexibility facilitates the entrance of ligands into the binding site and can also be involved in the communication between allosteric and orthosteric binding sites [66,67]. It should be noted that in any enzyme-ligand interaction, the enzyme undergoes a conformational change; therefore, variations in the enzyme’s flexibility might disrupt the function. Using the Dynamine server, each conjugate was evaluated to assess if the addition of CPP sequence to CPG2 interferes with the flexibility of zinc-binding amino acids (His^89^, Asp^119^, Glu^154^, Glu^178^, and His^363^). This might intervene in the ability of CPG2 attachment to the zinc molecules that are substantial for the detoxification of MTX. No changes were observed in His^89^, Asp^119^, Glu^154^, Glu^178^, and His^363^ flexibility scores in any of the conjugates. It can be concluded that the addition of CPPs had no interference with the CPG2 ligand-binding site.

### 2.9. Further Analyses for In Vivo Applications

In vivo administration of biotherapeutics comes with a new set of challenges. The objective of this section was to select the conjugates best suited for in vivo application (Table 9).

#### 2.9.1. Analyses of CPP-CPG2 and CPG2-CPP Conjugates

Some proteins might trigger mild to acute allergic responses and fatal anaphylactic shocks. Hence, the possibility of the allergenicity of a biotherapeutic should be explored. Hypersensitivity reactions were reported only in less than 1% of patients receiving glucarpidase [27]. Therefore, it should be investigated if the addition of a CPP sequence affects the probability of allergic reactions. Allergen FP V. 1.0 server evaluated CPG2 and all 140 CPP-CPG2/CPG2-CPP conjugates as probable non-allergens; thus, one can assume that the addition of these CPPs to the CPG2 sequence would not increase the enzyme’s allergenicity considerably.

One of the other notable characteristics in a therapeutic is immunogenicity. If the patient’s immune system recognizes a biotherapeutic as a threat, produced antibodies reduce the effectiveness of the medication after repeated use. Although glucarpidase does not result in hypersensitivity in most patients, studies have shown that anti-glucarpidase antibodies developed in 17% of patients receiving the medication for one or two doses [27]. In this regard, the fused CPP sequence preferably should not increase the antigenicity of CPG2 and if possible, alleviates the immunogenicity. Assessment of CPG2 and the top 70 CPPs conjugated to *N*- and C-terminal positions showed that CPG2 and all the conjugates had immunogenicity scores above 0.4, which might act as potential antigens. The addition of CPP resulted in a decrease in immunogenicity score in most conjugates. About 66% of *N*-terminal and 60% of C-terminal conjugates had an immunogenicity score lower than that of CPG2. *N*-terminal conjugates from peptides 1, 14, 47, 48, 49 and C-terminal conjugates from peptides 14, 19, 29, 48, and 70 displayed the least immunogenicity scores. Furthermore, it was concluded that the position of conjugations does not significantly affect the immunogenicity of CPP and CPG2 conjugates.

One of the concerns for protein therapeutics is their short biological half-lives. Proteins usually have fast degradation and clearance either by kidney filtration or liver metabolism. In the case of CPP-CPG2 conjugates, intracellular proteasomes might also expedite the frequency of administration. This shows the importance of the selection of the conjugates with optimal predicted half-lives. Using the ProtLifePred server, it was shown that all C-terminal conjugates have half-lives comparable to the unconjugated CPG2, while 31% of the N-terminal conjugates had half-lives longer than the unconjugated control. The rest of the conjugates had shorter or equal half-lives compared with un-conjugated CPG2 (Table S9).

#### 2.9.2. Analyses of top CPPs for In Vivo Application

Some proteins and peptides might have hemolytic toxicity towards red blood cells. Hemolysis is the premature loss of RBCs before their 120 days of the expected lifespan, which results in anemia. According to the HemoPI tool, CPPs named “Transportan 10 (TP10)” and “II” with the highest PROB scores might potentially have hemolysis effect after in vivo administration. “pVEC” mutants and “Retro - Tat (57–49)” had the lowest possibility of hemolysis. All the other CPPs had equal PROB scores relatively.

Another aspect that one should consider in the design of biotherapeutics is the undesired proinflammatory effect of proteins and peptides. For vaccines or immunotherapeutics, the ultimate goal is the activation of the immune system; however, for other proteins, proinflammatory effects such as T cell or B cell activation is undesirable. Proinflamm server checks the peptide sequence for some recognized proinflammatory epitopes. In this study, no inflammatory potential for the top 70 CPPs was detected.

As the aim of this study was to find the best candidates among experimentally validated CPPs for conjugations with CPG2, ToxinPred server was used to screen the top 70 CPPs using SVM based approach to identify toxic CPPs. Twenty percent of the top 70 CPPs were predicted to be toxic. All toxic peptides were derivatives of Crot (27–79), which originates from protein in snake venom. Unfortunately, no tool is available for the analysis of hemolytic activity and the proinflammatory effect of complete protein sequences.

### 2.10. Effect of Position of Conjugations on CPP-CPG2 and CPG2-CPP Conjugates

Throughout this study, the effect of position of the conjugation on the characteristics of all CPP-CPG2 and CPG2-CPP conjugates was analyzed using the unpaired t-test. The energy level of secondary structures at the 5′ mRNA’s initiation translation region, robustness in the protein’s 3D structures, higher helix and sheet content in the CPP region of the secondary structure after conjugation, and shorter half-folding times were all significantly improved in the N-terminal CPP-CPG2 conjugates compared with the C-terminal CPG2-CPP homologs. Regarding other features, including physiochemical properties, solubility, thermodynamic properties, probability of aggregation, backbone flexibility, allergenicity, and immunogenicity the position of conjugations was not significantly different between N- and C-terminal conjugates.

### 2.11. Most Promising CPP Candidates to Design CPP-CPG2/CPG2-CPP Conjugates

CPPs might influence the characteristics of the cargo protein of varying degrees. The ideal CPP for a conjugation would be the one that leads to the most favorable characteristics in the conjugate. As a result, “Bip 2”, “Bip 6”, “Bip 15”, “pAntp (45–58)”, “pAntp (46–58)”, “pAntp (47–58)”, “pAntp (48–58)”, “pAntp (49–58)”, “pAntp (51–58)”, “Ala44 substitution mutant of pAntp (43–58)”, “Ala52 substitution mutant of pAntp (43–58)”, “Tat (48–60)”, “Rev (34–50)”, and “Crot (27–39) derivative 7” were CPPs with the highest desirable evaluated characteristics in N-terminal conjugation to CPG2. “Bip 1”, “No.14–25”, and “No.14–35” were the most promising CPPs for the C-terminus (Table S10). These seventeen CPP candidates can be categorized into five families:

(1) HIV-Tat and Its Derivatives:

Tat peptide is derived from the transcription transactivator protein of the HIV-1 virus. Tat has been successfully used in conjugation with a variety of molecules such as peptides, proteins, liposomes, nanoparticles, and nucleic acids [68]. In this study, “Tat (48–60)” and one of its derivatives “Rev (34–50)” are on our final candidate list. This is in agreement with our recent findings, wherein we have demonstrated experimentally the efficient intracellular delivery of CPG2 conjugated to the TAT peptide [28]. Both native and denatured forms of the enzyme transduced efficiently into the HepG2 cells in a concentration- and time-dependent manner. We have observed that in cells pretreated with TAT-CPG2 protein, the viability in the presence of MTX increased up to 98.63% compared with the control group, which had a viability of about 44.37%. Hence, TAT-CPG2 was introduced as a strong protector of HepG2 cells against MTX-induced cell death.

(2) Penetratin Derivatives:

After the discovery of Tat, the penetratin peptide, which is derived from the amphiphilic *Drosophila* Antennapedia homeodomain (amino acids 43–58 of the homeodomain) was introduced as a CPP [69]. Currently, penetratin based therapeutics are being examined in vivo [70]. Eight CPPs from our final candidates including “pAntp (45–58)”, “pAntp (46–58)”, “pAntp (47–58)”, “pAntp (48–58)”, “pAntp (49–58)”, “pAntp (51–58)”, “Ala44 substitution mutant of pAntp (43–58)”, and “Ala52 substitution mutant of pAntp (43–58)” in the N-terminal conjugates are from this family.

(3) Crot (27–39) Derivatives:

Crotamin, a toxin found in snake venom, has two nuclear localization domains, named crot (2–18) and crot (27–39). Various derivatives of Crot (27–39) CPP with the original amino acid sequence of “KMDCRWRWKCCKK” are developed using amino acid substitution, deletion, or addition [49]. “Crot (27–39) derivative 7” from our candidate list belongs to this family.

(4) Cell-Penetrating Pentapeptides (CPP5s):

This group is designed according to the Bax-binding domain of Ku70 protein. Ku70 exerts cytoprotective effect by binding to Bax (a pro-apoptotic protein) and blocking its activation. Several cell-penetrating anti-apoptotic pentamer peptide sequences were designed based on the Bax-binding domain of Ku70. Subsequently, other CPP5s were developed by scrambling the sequence [71,72]. All five CPP5s are on our final candidates’ list, including “Bip 2”, “Bip 6” and “Bip 15” in the N-terminal conjugates, as well as “Bip 1”, in the C-terminal conjugates to CPG2. Among these CPP5s, “Bip 1”, “Bip 2”, and “Bip 6” have cytoprotective properties, while “Bip 15” is without any cytoprotective effects [37,73]. Therefore, for the elimination of MTX and cytoprotective purposes, “Bip 1”, “Bip 2”, and “Bip6” might have further beneficial effects in conjugation to CPG2.

(5) Peptide No. 14 Derivatives:

This family of peptides was invented using an in vitro virus library to design CPPs with high transduction efficacy. These CPPs displayed higher uptake efficacy compared with TAT in some cell lines [36]. Furthermore, these peptides are sequentially similar to tumor lineage-homing cell- penetrating peptides [35]. Peptides named “No.14–25” and “No.14–35” from our most promising candidates in C-terminal conjugates belongs to this group of peptides.

### 2.12. Analysis of Susceptibility to Human Proteases

Proteases are a group of enzymes that can cleave the peptide backbone of their target proteins. Some proteases are only able to recognize and cleave particular amino acid sequences known as cleavage sites. Proteolytic degradation of peptides and protein-based drugs is one of the major complications on the way of achieving optimum systemic administration of therapeutics [74]. Glucarpidase, like other peptide and protein-based biotherapeutics, is prone to proteolytic degradation. Although its stability in the blood is high enough to eliminate high concentrations of blood MTX, this might not be enough for successful application in ADEPT [75]. Therefore, several strategies have been applied to increase the resistance of glucarpidase to proteolytic enzymes, such as PEGylation, fusion with human serum albumin [76,77], and circular permutations [75].

Using the Prosperous server, CPG2 and the most promising conjugates (17 final conjugates) were evaluated against human proteases (Tables S11 and S12). The protease susceptibility of the conjugates is similar to the unconjugated CPG2, except for “Rev (34–50)-CPG2” that had a significant chance of being cleaved by “Kallikrein related peptidase 5”, while CPG2 and other CPP-CPG2 conjugates are expected to be resistant in the presence of the above-mentioned peptidase. It was observed that in some instances CPP-CPG2 conjugates had higher numbers of cleavage sites compared with CPG2 due to the addition of CPP sequence. However, the significant increase in cleavage sites has been observed for those proteases that target the unconjugated CPG2 even before the conjugation. Furthermore, some enzymes, for example, proprotein convertase 1 and 2, furin, and thrombin were able to cut inside the CPP sequence conjugated to CPG2. It could also be observed that the conjugation of CPP to CPG2 did not significantly decrease the protease susceptibility of CPG2.

### 2.13. Limitations of the Current Study

The mechanism of CPP translocation across plasma membranes is highly dependent on the type of cell line, the concentration of CPP, and the type of cargo [78]. Several CPPs can directly pass through the plasma membrane [79]; however, some others—especially those that are conjugated to macromolecules—penetrate through the endocytosis pathway. Endocytosis might result in the entrapment of the conjugate inside the endocytic organelles before arrival to the cytoplasmic or nuclear target [80]. Hence, the entrapment of CPPs and the corresponding conjugates inside the endosome is one of the limitations in the current computational and experimental studies. Although a bioinformatics method for the assessment of possible entrapment of CPP-protein conjugates is not available yet, using multivalent CPPs or CPP conjugation to a pH-Dependent Membrane Active Peptide (PMAP) are recommended to resolve inadequate CPP-protein endosomal release issues [79]. However, it was promising that in our experimental study conducted on the N-terminal conjugate of TAT-CPG2, the release of CPP-cargo conjugates from endosomes was high to display adequate pharmacological responses [28].

## 3. Materials and Methods

### 3.1. Primary Dataset Collection

Sequences of CPPs were retrieved from CPP site 2.0 (http://crdd.osdd.net/raghava/cppsite/), which keeps the records of experimentally validated CPPs [17]. After excluding cyclic peptides and CPPs that had un-natural residues or amino acids with D-conformations, 1155 unique CPPs remained. Although some of the excluded CPPs—CPPs with unnatural or D-conformation residues and cyclic CPPs—have shown promising results in experimental studies [81,82,83], current computational web servers are unable to analyze these sequences. Mature CPG2 amino acid sequence from *Pseudomonas* sp. strain RS-16 was retrieved from Uniprot (Uniprot ID # P06621) to build protein conjugates. The nucleotide sequence of the *cpg2* gene was then codon-optimized by the codon usage wrangler server (https://www.mrc-lmb.com.ac.uk/ms/methods/codon.html).

### 3.2. Penetration Prediction of CPPs

The uptake efficiency and respective prediction confidence for all 1155 CPPs was predicted using the CPPred-RF server (http://server.malab.cn/CPPred-RF/) [84]. Results of CPPred-RF sever analysis was narrowed down to 70 CPP candidates, which have the highest uptake efficiency with the prediction confidence of 0.9 or above.

### 3.3. mRNA Secondary Structure Prediction of cpg2 and cpp-cpg2/cpg2-cpp Conjugates

The nucleotide sequences of *cpg2* and top 70 *cpps* conjugated to either N- or C-terminus of *cpg2* gene in PET 14b expression vector (*cpp-cpg2*-pET14b) were used to determine the secondary structure and the minimum free energy of 5′-mRNA translation initiation region using the mfold online server (http://unafold.rna.albany.edu/?q=DINAMelt/Quickfold) [85]. To construct the N-terminal conjugates, 30 bases upstream of the start codon in PET 14b were fused to 30 initial bases of *cpps* linked to the codon-optimized *cpg2* gene. For the C-terminal conjugates, 30 bases upstream of the start codon were directly connected to the codon-optimized *cpg2*. PET 14b was selected due to our recent success in the expression of *cpg2* and *tat*- *cpg2* fusion constructs [28].

### 3.4. Prediction of Physiochemical Properties of CPPs and CPP-CPG2/CPG2-CPP Conjugates

Physiochemical properties of CPG2, N-terminal CPP-CPG2 and C-terminal CPG2-CPP conjugates, as well as the top 70 CPPs alone, including molecular weight (Mw), isoelectric point (pI), instability index, GRAVY, and aliphatic index were calculated using ProtParam tool (http://web.expasy.org/protparam/) [86].

### 3.5. Solubility Prediction of CPG2 and CPP-CPG2/CPG2-CPP Conjugates

The solubility of CPG2 and the top 70 CPPs linked to either *N*- or C-terminus of CPG2 was calculated using the ccSol server (http://s.tartaglialab.com/page/ccsol_group) [87].

### 3.6. Modeling 3D Structures of CPG2 and CPP-CPG2/CPG2-CPP Conjugates

FASTA sequences of CPG2 and the top 70 CPPs as *N*- and C-terminal conjugates to CPG2 were submitted to the Iterative Threading ASSEmbly Refinement (I-TASSER) program (https://zhanglab.ccmb.med.umich.edu/I-TASSER/) [88,89]. The most confident models (highest C-score value) derived from I-TASSER were uploaded to the PDBsum Generate EMBL-EBI (https://www.ebi.ac.uk/thornton-srv/databases/pdbsum/Generate.html) [90]. Subsequently, PDBsum (http://www.ebi.ac.uk/thornton-srv/databases/pdbsum) generated a Ramachandran plot for each model [91]. Ramachandran plots were analyzed for the most favored and additional allowed conformational regions. CPPs’ secondary structures after their conjugation to CPG2 in the models also were noted.

### 3.7. Thermodynamic Quantities of CPG2 and CPP-CPG2/CPG2-CPP Conjugates

PDB models of CPG2 and CPP-CPG2 N- and C-terminal conjugates were submitted to the SCOOP (http://babylone.ulb.ac.be/SCooP/k_query.php) [92] and FoldX (http://foldx.embl.de/) web servers [93]. SCOOP predicts the melting temperature (*T_m_*), changes in enthalpy (*ΔH_m_*), heat capacity upon folding (*ΔC_p_*), and the folding free energy at room temperature (*ΔG_r_*). Using FoldX Suite, some of the stability features including total free energy, side-chain hydrogen bond (Side H bond), solvation polar (solp), and Van der Waals clashes (VdW clash) energies were calculated.

### 3.8. Prediction of Aggregation Possibility of CPG2 and CPP-CPG2/CPG2-CPP Conjugates

FASTA sequences of CPG2 and the top 70 CPPs linked to the N- and C-terminus of CPG2 were submitted to PASTA 2.0 (http://protein.bio.unipd.it/pasta2/) [94] and Aggrescan (http://bioinf.uab.es/aggrescan/) [63] servers to compare aggregation possibility of conjugated *versus* un-conjugated CPG2 protein. PASTA 2.0 analyzes segments that are more likely to form fibrillar aggregates, and Aggrescan predicts the number of hot spots for aggregation in a sequence.

### 3.9. Folding Rate of CPG2 and CPP-CPG2/CPG2-CPP Conjugates

Sequences of CPG2 and the top 70 CPPs linked to the N- and C-terminus of CPG2 were submitted to the Foldrate server (http://www.csbio.sjtu.edu.cn/bioinf/FoldRate/) [95]. Foldrate provides an estimation on the time needed for proteins to fold into their tertiary structure by predicting ln(*K*_f_) constant of folding and half-folding time.

### 3.10. Backbone Flexibility of CPG2 and CPP-CPG2/CPG2-CPP Conjugates

Amino acid sequences of CPG2 and both N- and C-terminal conjugates of the top 70 CPPs and CPG2 were analyzed using the DynaMine server (http://dynamine.ibsquare.be/) [96]. DynaMine predicts the flexibility of each amino acid in a protein sequence by attributing values between 0–1 to each residue. Zero accounts for complete flexibility and 1 for complete rigidity.

### 3.11. Further Analyses for In Vivo Application

#### 3.11.1. Analyses of CPP-CPG2 and CPG2-CPP Conjugates

Sequences of 70 top CPP-CPG2 N-terminal and CPG2-CPP C-terminal conjugates were analyzed using AllergenFP V 1.0 (http://ddg-pharmfac.net/AllergenFP/) [97] to predict their potential allergenicity. Sequences of both *N*- and C-terminal conjugates of the top 70 CPPs and CPG2 were analyzed for potential immunogenicity by the VaxiJen V2.0 server (http://www.ddgpharmfac.net/vaxijen/VaxiJen/VaxiJen.html) [98] considering bacteria as selected target organism. The half-life of CPG2 conjugated to the top 70 CPPs (both N- and C-terminal conjugates) was calculated using the ProtLifePred server (http://protein-n-end-rule.leadhoster.com/) in *E. coli* as the expression system. ProtLifePred server estimates the half-life of protein sequences based on the N-end rule considering ubiquitination [99,100].

#### 3.11.2. Analyses of CPPs

The top 70 CPPs were analyzed for their possible RBC lysis effect after in vivo administration by the HemoPI server (http://crdd.osdd.net/raghava/hemopi/) [101]. HemoPI server analyses submitted sequences using SVM based approach to predict their hemolysis potency by assigning each query a PROB score that ranges between 0-1. Zero is an indication of the lowest possibility of being hemolytic. In this study, the PROB score was set on 0.5 to determine hemolytic toxicity for each CPP. Potential toxicity of the top 70 CPPs was evaluated by ToxinPred server (http://crdd.osdd.net/raghava/toxinpred/multi_submit.php) [102], using the server’s default threshold (zero). Top 70 CPPs were checked for pro-inflammatory effect using the ProInflam server (http://metagenomics.iiserb.ac.in/proinflam/index.html) [103].

### 3.12. Evaluation of CPP-CPG2/CPG2-CPP Conjugates Susceptibility to Human Proteases

The susceptibility of the most promising CPP-CPG2 and CPG2-CPP conjugates to human proteases was evaluated using the Prosperous web server (http://prosperous.erc.monash.edu/) [104,105]. Out of 90 proteases available on the Prosperous server, 51 were human proteases according to MEROPS: the peptidase database [106]. These human proteases are categorized into four families, including aspartic proteases like cathespsin D and E, cysteine proteases like caspase 1 and 3, metalloproteases like matrix metallopeptidase 1,2, 3, and serine proteases like thrombin and plasmin. 

**Scheme 1:**
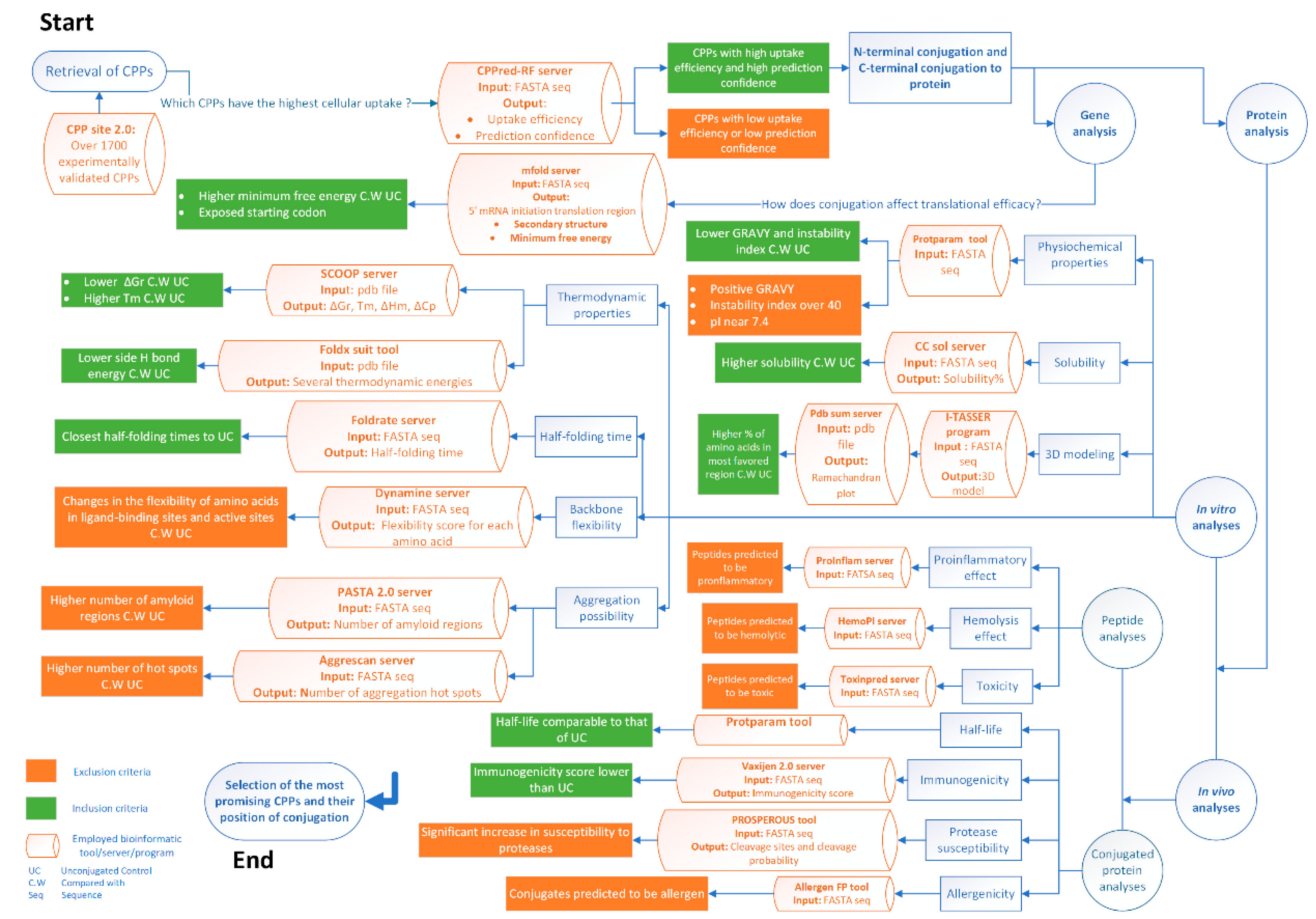
A summary of the proposed workflow to select the most promising CPPs in conjugation with a protein.

Prosperous assigns each cleavage site a probability score between 0.0 and 1.0. Zero is considered as the lowest possibility and 1.0 is estimated as the highest possibility of a position being recognized and cleaved by a protease. In this analysis, the FASTA sequences of CPG2 and 17 most promising CPP-CPG2/CPG2-CPP conjugates were submitted to Prosperous. A probability score of 0.700 and higher was considered significant for the susceptibility to human proteases. An overview of analyses employed in this study is shown as a bioinformatics flowchart below (Scheme 1).

### 3.13. Statistical Analyses

Unpaired t-test was used to test the significance between N-terminal (CPP-CPG2) and C-terminal (CPG2-CPP) values using GraphPad Prism 8 software (GraphPad Software, Inc. San Diego, CA, USA). *p* < 0.05 indicated statistical significance.

## 4. Conclusions

Within this study, characterizing a high number of CPPs highlighted crucial factors necessary for the rational design of a CPP-protein chimera. This study presented a bioinformatics workflow applicable as a comprehensive approach useful to select CPP-cargo constructs for any therapeutic application. The addition of CPPs resulted in a higher probability of translation of mRNA into protein in about 74% of the *N*-terminal conjugates. Structurally more than 70% of the CPPs had α-helix or β-sheet conformations in their secondary structure after conjugations, which results in higher penetration than for non-fused cargo. Addition of CPPs did not cause any significant negative changes regarding the stability of the target protein (thermodynamic stability and resistance to human proteases). Except for a few CPPs which might result in higher aggregation of the chimera, analysis of the physiochemical characteristics showed that the dominant number of CPPs linked to the cargo did not have any negative influence on the properties. Conjugation of CPPs did not negatively interfere with the solubility, even improved it. Computational analyses showed that allergenicity is not affected negatively and does not lead to limitations for injectable formulations. Flexibility analysis showed that in most conjugates, the addition of CPPs caused no major alterations in the overall enzyme flexibility. In all conjugates, the flexibility of the ligand-binding site after the addition of a CPP remained unchanged. However, stronger servers are needed for evaluation of the RBC hemolysis and pro-inflammatory effect of the peptide-protein conjugate. Concerning the position of conjugations, the N-terminal linkage is preferred for the construction of CPP-CPG2 conjugates than the C-terminus CPG2-CPP homologs. The approach presented in this investigation is not limited to the CPP-glucarpidase case study but is generally applicable to any CPP-protein conjugate design. Hence, our study provides a platform for further in vitro and in vivo investigations, which should be considered regarding the advantages of computational analyses before designing any covalently conjugated CPP-protein construct.

## Figures and Tables

**Table 1:** Results of CPPred-RF server analysis for 70 cell-penetrating peptides (CPPs) that had high uptake efficiency with prediction confidence of above 0.9.

Peptide No.	CPPs’ Names	Amino Acid Sequence	Cell-Penetrating or Not	Prediction Confidence ^1^	Uptake Efficiency	Prediction Confidence ^2^
1	Transportan 10 (TP10)	AGYLLGKINLKALAALAKKIL	Cell-penetrating	0.98	High	1.00
2	Ala43 substitution mutant of pAntp (43–58)	AQIKIWFQNRRMKWKK	Cell-penetrating	0.95	High	0.96
3	Crot (27–39) derivative 1	CRFRFKCCKK	Cell-penetrating	0.96	High	0.98
4	Crot (27–39) derivative 2	CRFRWKCCKK	Cell-penetrating	0.96	High	0.99
5	Crot (27–39) derivative 3	CRWRFKCCKK	Cell-penetrating	0.96	High	1.00
6	CyLoP–1	CRWRWKCCKK	Cell-penetrating	0.95	High	1.00
7	Crot (27–39) derivative 4	CRWRWKCGCKK	Cell-penetrating	0.92	High	0.99
8	Crot (27–39) derivative 5	DCRWRWKCCKK	Cell-penetrating	0.82	High	0.99
9	pAntp (49–58)	FQNRRMKWKK	Cell-penetrating	0.84	High	0.91
10	Tat (48–60)	GRKKRRQRRRPPQ	Cell-penetrating	0.97	High	0.94
11	pAntp (45–58)	IKIWFQNRRMKWKK	Cell-penetrating	0.93	High	0.91
12	Bip15	IPMLK	Cell-penetrating	0.56	High	0.92
13	pAntp (47–58)	IWFQNRRMKWKK	Cell-penetrating	0.89	High	0.97
14	II	KALAALLKKLAKLLAALK	Cell-penetrating	1.00	High	0.93
15	Crot (27–39) derivative 6	KCCKWRWRCK	Cell-penetrating	0.95	High	0.94
16	Crot (27–39) derivative 7	KCGCRWRWKCGCKK	Cell-penetrating	0.95	High	0.90
17	Crot (27–39) derivative 8	KCRWRWKCCKK	Cell-penetrating	0.95	High	0.98
18	Crot (27–39) derivative 9	KDCRWRWKCCKK	Cell-penetrating	0.78	High	0.99
19	pAntp (46–58)	KIWFQNRRMKWKK	Cell-penetrating	0.93	High	0.96
20	7	KLWMRWWSPTTRRYG	Cell-penetrating	0.98	High	0.93
21	No.14–2	KLWMRWYSATTRRYG	Cell-penetrating	0.98	High	0.97
22	No.14	KLWMRWYSPTTRRYG	Cell-penetrating	0.98	High	0.96
23	No.14–7	KLWMRWYSPWTRRYG	Cell-penetrating	0.96	High	0.92
24	Crot (27–39)	KMDCRWRWKCCKK	Cell-penetrating	0.80	High	0.94
25	Crot (27–39) derivative 10	KMDCRWRWKCKK	Cell-penetrating	0.78	High	0.95
26	Crot (27–39) derivative 11	KMDCRWRWKCSKK	Cell-penetrating	0.82	High	0.95
27	Crot (27–39) derivative 12	KMDCRWRWKSCKK	Cell-penetrating	0.83	High	0.95
28	pVEC mutant 1	LLIILRARIRKQAHAHSK	Cell-penetrating	0.98	High	0.90
29	pVEC mutant 2	LLIILRRAIRKQAHAHSK	Cell-penetrating	0.98	High	0.95
30	pVEC mutant 3	LLIILRRRIRAQAHAHSK	Cell-penetrating	0.98	High	0.94
31	Crot (27–39) derivative 13	MDCRWRWKCCKK	Cell-penetrating	0.79	High	0.93
32	ARF (1-22)	MVRRFLVTLRIRRACGPPRVRV	Cell-penetrating	0.88	High	0.93
33	M918	MVTVLFRRLRIRRACGPPRVRV	Cell-penetrating	0.90	High	0.94
34	pAntp (51–58)	NRRMKWKK	Cell-penetrating	0.90	High	0.91
35	pAntp (44–58)	QIKIWFQNRRMKWKK	Cell-penetrating	0.96	High	0.92
36	pAntp (50–8)	QNRRMKWKK	Cell-penetrating	0.88	High	0.96
37	Ala44 substitution mutant of pAntp (43–58)	RAIKIWFQNRRMKWKK	Cell-penetrating	1.00	High	0.99
38	PDX -1-PTD	RHIKIWFQNRRMKWKK	Cell-penetrating	0.99	High	0.92
39	No.14–25	RLFMRFYSPTTRRYG	Cell-penetrating	0.95	High	0.93
40	No.14–17	RLWMRWASPTTRRYG	Cell-penetrating	0.99	High	0.96
41	No.14–18	RLWMRWYAPTTRRYG	Cell-penetrating	0.98	High	0.98
42	No.14–20	RLWMRWYSPATRRYG	Cell-penetrating	0.99	High	1.00
43	No.14–21	RLWMRWYSPTARRYG	Cell-penetrating	0.99	High	1.00
44	No.14–35	RLWMRWYSPTTRRYA	Cell-penetrating	0.98	High	0.98
45	No.14–1	RLWMRWYSPTTRRYG	Cell-penetrating	0.99	High	0.98
46	30	RLYMRYYSPTTRRYG	Cell-penetrating	0.97	High	0.93
47	Ala45 substitution mutant of pAntp (43–58)	RQAKIWFQNRRMKWKK	Cell-penetrating	0.98	High	0.98
48	Ala46 substitution mutant of pAntp (43–58)	RQIAIWFQNRRMKWKK	Cell-penetrating	0.98	High	0.91
49	Ala47 substitution mutant of pAntp (43–58)	RQIKAWFQNRRMKWKK	Cell-penetrating	0.99	High	0.99
50	Ala48 substitution mutant of pAntp (43–58)	RQIKIAFQNRRMKWKK	Cell-penetrating	1.00	High	0.94
51	Ala49 substitution mutant of pAntp (43–58)	RQIKIWAQNRRMKWKK	Cell-penetrating	1.00	High	0.98
52	Ala50 substitution mutant of pAntp (43–58)	RQIKIWFANRRMKWKK	Cell-penetrating	0.99	High	0.99
53	pAntpHD (Pro50)	RQIKIWFPNRRMKWKK	Cell-penetrating	0.99	High	0.96
54	Ala51 substitution mutant of pAntp (43–58)	RQIKIWFQARRMKWKK	Cell-penetrating	0.99	High	0.94
55	Ala52 substitution mutant of pAntp (43–58)	RQIKIWFQNARMKWKK	Cell-penetrating	0.95	High	0.92
56	Met-Arg	RQIKIWFQNMRRKWKK	Cell-penetrating	1.00	High	0.93
57	Ala54 substitution mutant of pAntp (43–58)	RQIKIWFQNRRAKWKK	Cell-penetrating	0.99	High	0.97
58	Penetratin	RQIKIWFQNRRMKWKK	Cell-penetrating	1.00	High	0.97
59	Retro - Tat (57–49)	RRRQRRKKR	Cell-penetrating	1.00	High	0.90
60	R6	RRRRRR	Cell-penetrating	1.00	High	0.91
61	R9	RRRRRRRRR	Cell-penetrating	1.00	High	0.91
62	Crot (27–39) derivative 14	RWRWKCCKK	Cell-penetrating	0.91	High	0.97
63	Crot (27–39) derivative 15	SRWRWKCCKK	Cell-penetrating	0.94	High	0.93
64	Rev (34–50)	TRQARRNRRRRWRERQR	Cell-penetrating	0.98	High	0.90
65	HIV-1 Rev (34–50)	TRQARRNRRRRWRERQRGC	Cell-penetrating	0.96	High	0.90
66	Bip6	VPALK	Cell-penetrating	0.74	High	0.96
67	Bip1	VPMLK	Cell-penetrating	0.57	High	0.96
68	Bip2	VPTLK	Cell-penetrating	0.67	High	0.99
69	Bip16	VPTLQ	Cell-penetrating	0.60	High	0.91
70	pAntp (48–58)	WFQNRRMKWKK	Cell-penetrating	0.84	High	0.97

^1^ Prediction confidence of cell penetration. ^2^ Prediction confidence of uptake efficiency.

**Table 2:** Experimental data on the uptake efficiency of top 70 CPPs.

Category	Names of CPPs	Experimental Uptake Efficiency	References
**Crot (27–39) and its derivatives**	CyLoP-1	High, higher than D-Tat peptide, penetratin, and D-R8	[29]
	Crot (27–39)	78% of CyLoP1	
	Crot (27–39) derivative 1	63% of CyLoP1	
	Crot (27–39) derivative 2	66% of CyLoP1	
	Crot (27–39) derivative 3	61% of CyLoP1	
	Crot (27–39) derivative 4	59% of CyLoP1	
	Crot (27–39) derivative 5	47% of CyLoP1	
	Crot (27–39) derivative 6	42% of CyLoP1	
	Crot (27–39) derivative 7	75% of CyLoP1	
	Crot (27–39) derivative 8	39% of CyLoP1	
	Crot (27–39) derivative 9	26% of CyLoP1	
	Crot (27–39) derivative 10	79% of CyLoP1	
	Crot (27–39) derivative 11	58% of CyLoP1	
	Crot (27–39) derivative 12	29% of CyLoP1	
	Crot (27–39) derivative 13	83% of CyLoP1	
	Crot (27–39) derivative 14	37% of CyLoP1	
	Crot (27–39) derivative 15	46% of CyLoP1	
**Penetratin and its derivatives**	Penetratin/pAntp (43–58)	Lower than polyarginines but higher than Tat peptide and transportan	[30]
	pAntp (44–58)	85% of pAntp (43–58)	[31]
	pAntp (45–58)	95% of pAntp (43–58)	
	pAntp (46–58)	65% of pAntp (43–58)	
	pAntp (47–58)	50% of pAntp (43–58)	
	pAntp (48–58)	55% of pAntp (43–58)	
	pAntp (49–58)	65% of pAntp (43–58)	
	pAntp (50–58)	60% of pAntp (43–58)	
	pAntp (51–58)	60% of pAntp (43–58)	
	Ala43 substitution mutant of pAntp (43–58)	90% of pAntp (43–58)	
	Ala44 substitution mutant of pAntp (43–58)	65% of (pAntp) (43–58)	
	Ala45 substitution mutant of pAntp (43–58)	80% of pAntp (43–58)	
	Ala46 substitution mutant of pAntp (43–58)	50% of pAntp (43–58)	
	Ala47 substitution mutant of pAntp (43–58)	55% of pAntp (43–58)	
	Ala48 substitution mutant of pAntp (43–58)	65% of pAntp (43–58)	
	Ala49 substitution mutant of pAntp (43–58)	90% of pAntp (43–58)	
	Ala50 substitution mutant of pAntp (43–58)	90% of pAntp (43–58)	
	Ala51 substitution mutant of pAntp (43–58)	60% of pAntp (43–58)	
	Ala52 substitution mutant of pAntp (43–58)	45% of pAntp (43–58)	
	Ala54 substitution mutant of pAntp (43–58)	90% of pAntp (43–58)	
	Met-Arg	Not available	[32]
	pAntpHD (Pro50)	High Comparable to pAntp (43–58)	[33]
**CPP derived from PDX-1 protein**	PDX-1-PTD	Not available	[34]
**Tumor lineage-homing CPPs**	7	Not available	[35]
**Peptide 14 and its derivatives**	No.14	Higher than Tat peptide	[36]
	No.14–1	Higher than Tat and peptide No. 14	
	No.14–2	Higher than Tat peptide	
	No.14–7	Higher than Tat and peptide No. 14	
	No.14–17	Equal to Peptide No. 14	
	No.14–18	Higher than Peptide 14 and equal to peptide 14–1	
	No.14–20	Higher than Peptide 14 and equal to peptide 14–1	
	No.14–21	Higher than Peptide 14 and equal to peptide 14–1	
	No.14–25	Equal to peptide 14	
	No.14–30	Higher than peptide 14 and lower than peptide 14–1	
	No.14–35	Higher than Peptide 14 and equal to peptide 14–1	
**Cell-penetrating penta peptides** **(CPP5s)**	Bip1	High About 97% of KLPVM	[37]
	Bip2	About 61% of KLPVM)	
	Bip6	About 71% of KLPVM	
	Bip15	Not available	
	Bip16	About 70% KLPVM	
**pVEC (CPP derived from murine vascular endothelial cadherin)**	pVEC mutant 1	Comparable to pVEC	[38]
	pVEC mutant 2	Higher than pVEC	
	pVEC mutant 3	Comparable to pVEC	
**Tat peptide and its derivatives**	Tat (48–60)	High Lower than polyarginine and penetratin but equal to transportan	[30,39]
	Rev (34–50)	High Comparable to Tat (48–60)	[40]
	HIV-1 Rev (34–50)	2.5-6.6 times more than Tat (48–60)	[41]
	Retro - Tat (57–49)	High Compared to Tat (49–57)	[42]
**Polyarginines**	R6	Lower than R9	[42]
	R9	High	
**Transportan peptide derivative**	Transportan 10 (TP10)	High	[43]
**CPPs derived from tumor suppressor protein p14ARF**	ARF(1–22)	High Comparable to TP10	[43]
	M918	Higher than Penetratin	[44]
**α-helical amphipathic CPPs**	II	High	[45]

**Table 3:** Top 70 CPPs’ secondary structure prediction after conjugation with CPG2.

Peptide Number	CPPs’ Names	N-Terminal Conjugation with CPG2		C-Terminal Conjugation with CPG2	
		Secondary Structure after Conjugation with CPG2 (C: coil, S: Sheet, H: Helix)	Percentage of Amino Acids with Helix and Sheet Configuration	Secondary Structure after Conjugation with CPG2 (C: coil, S: sheet, H: helix)	Percentage of Amino Acids with Helix and Sheet Configuration
**1**	Transportan 10 (TP10)	CCSSSSSCHHHHCCCCCHHHC	57.1	CCCCCCCCCCHHHHHHHHHCC	42.9
**2**	Ala43 substitution mutant of pAntp (43–58)	CCHHHHHHHCCCCCHH	56.2	HHHHHHHHHHHHHHCC	87.5
**3**	Crot (27–39) derivative 1	CSSSSSHHHH	90.0	CCCSSSSCCC	40.0
**4**	Crot (27–39) derivative 2	CSSSSSSHHH	90.0	CCCSSSSCCC	40.0
**5**	Crot (27–39) derivative 3	CSSSSSSCHH	80.0	CCHHHSSCCC	50.0
**6**	CyLoP-1	CSSSHHHHHH	90.0	CCCCCCCCCC	0.0
**7**	Crot (27–39) derivative 4	CCCCCCCCCCH	9.1	CCCCCCCCCCC	0.0
**8**	Crot (27–39) derivative 5	CCSSSSHHHHH	81.8	CHHHHHHCCCC	54.5
**9**	pAntp (49–58)	CCCCHHHHHH	60.0	CCCCCCCCCC	0.0
**10**	Tat (48–60)	CCCCCCCCCCCCC	0.0	CCCCCCCCCCCCC	0.0
**11**	pAntp (45–58)	CCSSSCCCCCCCHH	35.7	CCCCCCCCCCCCCC	0.0
**12**	Bip15	CCCCC	0.0	CCCCC	0.0
**13**	pAntp (47–58)	CCCCCCCCCCHH	16.7	CCCCCCCCCCCC	0.0
**14**	II	CHHHHHHHHHHHHHHHHH	94.4	HHHHHHHHHHHHHHHHCC	88.9
**15**	Crot (27–39) derivative 6	CCCCCCCCCC	0.0	CCCCCCCCCC	0.0
**16**	Crot (27–39) derivative 7	CCCCCCCCCCCCCH	7.1	CCHHHHHHHCCCCC	50.0
**17**	Crot (27–39) derivative 8	CCSSSSSCHHH	27.3	CCCCCCCCCCC	0.0
**18**	Crot (27–39) derivative 9	CCCSSSSHHHHH	41.7	CCCCCCCHHCCC	16.7
**19**	pAntp (46–58)	CCCCCCCCCCCHH	15.4	CCCCCHHHHHHCC	46.2
**20**	7	CCCSSSCCCCCCCCH	26.7	CCCCCCCCCCCCCCC	0.0
**21**	No.14–2	CHHHHHHHHHHHCCC	73.3	HHHCCCCHHHHCCCC	46.7
**22**	No.14	CCSSSSSCCCCCCHH	53.3	CCCCCCCCCCCCCCC	0.0
**23**	No.14–7	CCHHHHHCCCCHHHH	60.0	CCCCCCCCCCCCCCC	0.0
**24**	Crot (27–39)	CCCSSSSSHHHHH	76.9	CCCCCHHHHHCCC	38.5
**25**	Crot (27–39) derivative 10	CCCCSSHHHHHH	66.7	CCCCCCCCCCCC	0.0
**26**	Crot (27–39) derivative 11	CCCCCCSSCCCHH	30.8	CCCCCCCCCCCCC	0.0
**27**	Crot (27–39) derivative 12	CCCSSSSHHHHHH	76.9	CCCCHHHHHHCCC	46.2
**28**	pVEC mutant 1	CHHHHHHHHHCCCCCCCH	55.5	CCHHHHHHHHHHHHCCCC	66.7
**29**	pVEC mutant 2	CCSSSHHHHHCCCCCCCC	44.4	HHHHHHHHHHHCCCCCCC	61.1
**30**	pVEC mutant 3	CSSSHHHHHHHCCCCCCH	61.1	CCHHHHHHHHHHHCCCCC	61.1
**31**	Crot (27–39) derivative 13	CCCSSSSHHHHH	75.0	CCCCHHHHHCCC	41.7
**32**	ARF(1–22)	CCCHHHHHHHHHHHHCCCCCCC	54.5	HHHHHHHHHHHCCCCCCCCCCC	50.0
**33**	M918	CCSSHHHHHHHHHHCCCCCCCC	54.5	CCHHHHHHHHCCCCCCCCCCCC	36.4
**34**	pAntp (51–58)	CCCCCCHH	25.0	CCCCCCCC	0.0
**35**	pAntp (44–58)	CCCHHHHCCCCCCHH	40.0	HHHHHHHHHCCCCCC	60.0
**36**	pAntp (50–58)	CCCCCCCHH	22.2	CCCCCCCCC	0.0
**37**	Ala44 substitution mutant of pAntp (43–58)	CCCCHHHCCCCCCCHH	31.2	HHHHHHHHCCHHHCCC	68.8
**38**	PDX -1-PTD	CCCHHHHCCCCCCCHH	37.5	HHHHHHHHHHHCCCCC	68.8
**39**	No.14–25	CSSSSSCCCHHHHHH	73.3	CHHCCCCCCCCCCCC	13.3
**40**	No.14–17	CSSSSCCCCCCHHHH	53.3	HHHHCCCCCCCCCCC	26.7
**41**	No.14–18	CCSSSSSCCCCCCCC	33.3	HHHHHHHCCCCCCCC	46.7
**42**	No.14–20	CCCCCCCCCCCCCCH	6.7	CCCCCCCCCCCCCCC	0.0
**43**	No.14–21	CCSSSSSSCCCHHHH	66.7	HHHHHHHHHHHHHCC	86.7
**44**	No.14–1135	CCSSSSSCCCCCCCC	33.3	CCCCCCCCCCCCCCC	0.0
**45**	No.14–1	CCCSSSCCCCCCCHH	33.3	CCCCCCCCCCCCCCC	0.0
**46**	30	CCCSSSCCCHHHHCC	46.7	CCCCCCCCCCCCCCC	0.0
**47**	Ala45 substitution mutant of pAntp (43–58)	CCCSSSSCCCCCCCHH	37.5	HHHHHHHHHHHHHCCC	81.3
**48**	Ala46 substitution mutant of pAntp (43–58)	CCSSSSSCCCCCCCHH	43.7	HHHHHHHCCCHHHHCC	68.8
**49**	Ala47 substitution mutant of pAntp (43–58)	CCHHHCCCCCCCCCHH	31.2	HHHHHHHHHHCCCCCC	62.5
**50**	Ala48 substitution mutant of pAntp (43–58)	CCHHHHHHHCCCCCCC	43.7	CCCCHHHCHHHHHCCC	50.0
**51**	Ala49 substitution mutant of pAntp (43–58)	CCCSSCCCCCCCCCHH	25	HHCCHHHHCCCHHCCC	50.0
**52**	Ala50 substitution mutant of pAntp (43–58)	CCSSSSSCCCCCCCHH	43.7	HHHHHHHHHHHHCCCC	87.5
**53**	pAntpHD (Pro50)	CCCCCCCCCCCCCCHH	12.5	HHHHHHCCCCHHHCCC	68.8
**54**	Ala51 substitution mutant of pAntp (43–58)	CCCSSSSCCCCCCCCH	31.2	CCCCCCHHHHHHHHCC	50.0
**55**	Ala52 substitution mutant of pAntp(43–58)	CCHHHHHHHHHHHCCC	68.7	CCCCCHHHCCHHHCCC	37.5
**56**	Met-Arg	CCHHHHHHHHHHHHHH	87.5	HHHHHHHHHHHHHHCC	87.5
**57**	Ala54 substitution mutant of pAntp(43–58)	CSSSHHHCCCCCCCHH	50.0	HHHHHHHHCCHHHHCC	75.0
**58**	Penetratin	CCSSSSSCCCCCCCCH	37.5	HHHHHHHHHHHHHHCC	87.5
**59**	Retro - Tat (57–49)	CCCCCCCCC	0.0	CCCCCCCCC	0.0
**60**	R6	CCHHHH	66.7	CCCCCC	0.0
**61**	R9	CCCCCCCCC	0.0	CCCCHHCCC	22.2
**62**	Crot (27–39) derivative 14	CSSHHHHHH	88.9	CCCSSSCCC	33.3
**63**	Crot (27–39) derivative 15	CCSSSHHHHH	80.0	CCCCCCCCCC	0.0
**64**	Rev (34–50)	CCCCCCCCCCCCCCCCC	0.0	CCCCCCCCCCCCCCCCC	0.0
**65**	HIV-1 Rev (34–50)	CCCCCCCCCHHHHHHHCCC	36.8	CCCCCCCCCCCCCCCCCCC	0.0
**66**	Bip6	CCCCC	0.0	CCCCC	0.0
**67**	Bip1	CCCCC	0.0	CCCCC	0.0
**68**	Bip2	CCCCC	0.0	CCCCC	0.0
**69**	Bip16	CCCCC	0.0	CCCCC	0.0
**70**	pAntp (48–58)	CCCCCCCCCHH	18.2	CCCCCCCCCCC	0.0

**Table 4:** Physiochemical properties of CPP-CPG2 *N*-terminal conjugates.

Peptide Number	Name of the Conjugate	Number of Amino Acids	Mw (Da)	pI	Instability Index	Aliphatic Index	GRAVY
**1**	Transportan 10 (TP10)-CPG2	414	43860.19	8.37	23.99	97.20	−0.116
**2**	Ala43 substitution mutant of pAntp (43-58)-CPG2	409	43839.07	8.82	25.71	91.69	−0.218
**3**	Crot (27–39) derevative 1-CPG2	403	42996.11	8.48	24.58	90.87	−0.187
**4**	Crot (27–39) derevative 2-CPG2	403	43035.15	8.48	26.37	90.87	−0.196
**5**	Crot (27–39) derevative 3-CPG2	403	43035.15	8.48	26.00	90.87	−0.196
**6**	CyLoP-1-CPG2	403	43074.18	8.48	27.79	90.87	−0.205
**7**	Crot (27–39) derevative 4-CPG2	404	43131.24	8.48	27.75	90.64	−0.206
**8**	Crot (27–39) derevative 5-CPG2	404	43189.27	8.21	27.75	90.64	−0.214
**9**	pAntp (49–58)-CPG2	403	43099.16	8.65	26.37	90.87	−0.228
**10**	Tat (48–60)-CPG2	406	43396.46	9.08	31.77	90.20	−0.279
**11**	pAntp (45–58)-CPG2	407	43639.86	8.82	25.79	91.89	−0.215
**12**	Bip15–CPG2	398	42278.25	6.56	24.66	93.97	−0.159
**13**	pAntp (47–58)-CPG2	405	43398.53	8.65	26.29	91.38	−0.218
**14**	II-CPG2	411	43554.90	8.64	23.83	97.20	−0.121
**15**	Crot (27–39) derevative 6-CPG2	403	43074.18	8.48	26.37	90.87	−0.205
**16**	Crot (27–39) derevative 7-CPG2	407	43419.60	8.61	27.62	89.98	−0.209
**17**	Crot (27–39) derevative 8-CPG2	404	43202.36	8.66	27.75	90.64	−0.215
**18**	Crot (27–39) derevative 9-CPG2	405	43317.45	8.47	27.70	90.42	−0.223
**19**	pAntp (46–58)-CPG2	406	43526.70	8.82	26.04	91.16	−0.227
**20**	7-CPG2	408	43701.80	8.39	28.82	90.71	−0.215
**21**	No.14–2-CPG2	408	43652.73	8.38	27.74	90.96	−0.208
**22**	No.14-CPG2	408	43678.77	8.38	28.82	90.71	−0.216
**23**	No.14–7-CPG2	408	43763.88	8.38	28.38	90.71	−0.216
**24**	Crot (27–39)-CPG2	406	43448.64	8.47	28.46	90.20	−0.217
**25**	Crot (27–39) derevative 10-CPG2	405	43345.50	8.53	28.51	90.42	−0.224
**26**	Crot (27–39) derevative 11-CPG2	406	43432.58	8.53	28.46	90.20	−0.226
**27**	Crot (27–39) derevative 12-CPG2	406	43432.58	8.53	29.27	90.20	−0.226
**28**	pVEC mutant 1-CPG2	411	43802.04	8.66	25.76	95.52	−0.169
**29**	pVEC mutant 2-CPG2	411	43802.04	8.66	27.15	95.52	−0.169
**30**	pVEC mutant 3-CPG2	411	43830.05	8.67	27.97	95.52	−0.171
**31**	Crot (27–39) derevative 13-CPG2	405	43320.46	8.17	27.70	90.42	−0.208
**32**	ARF(1–22)-CPG2	415	44329.74	8.93	28.88	94.10	−0.158
**33**	M918-CPG2	415	44329.74	8.93	28.88	94.10	−0.158
**34**	pAntp (51–58)-CPG2	401	42823.85	8.65	26.45	91.32	−0.227
**35**	pAntp (44–58)-CPG2	408	43767.99	8.82	25.75	91.67	−0.223
**36**	pAntp (50–58)-CPG2	402	42951.98	8.65	26.41	91.09	−0.235
**37**	Ala44 substitution mutant of pAntp (43–58)-CPG2	409	43867.13	8.95	25.71	91.69	−0.221
**38**	PDX -1-PTD-CPG2	409	43933.19	8.95	27.26	91.44	−0.233
**39**	No.14–25-CPG2	408	43628.71	8.39	27.26	90.71	−0.199
**40**	No.14–17-CPG2	408	43614.68	8.40	28.66	90.96	−0.210
**41**	No.14–18-CPG2	408	43690.78	8.39	29.00	90.96	−0.211
**42**	No.14–20-CPG2	408	43676.75	8.39	29.50	90.96	−0.211
**43**	No.14–21-CPG2	408	43676.75	8.39	29.02	90.96	−0.211
**44**	No.14–35-CPG2	408	43720.81	8.39	30.02	90.96	−0.212
**45**	No.14–1-CPG2	408	43706.78	8.39	29.02	90.71	−0.217
**46**	30-CPG2	408	43660.71	8.37	27.65	90.71	−0.219
**47**	Ala45 substitution mutant of pAntp (43–58)-CPG2	409	43882.10	8.95	26.39	90.73	−0.240
**48**	Ala46 substitution mutant of pAntp (43–58)-CPG2	409	43867.09	8.83	26.60	91.69	−0.220
**49**	Ala47 substitution mutant of pAntp (43–58)-CPG2	409	43882.10	8.95	26.39	90.73	−0.240
**50**	Ala48 substitution mutant of pAntp (43–58)-CPG2	409	43809.00	8.95	26.19	91.69	−0.227
**51**	Ala49 substitution mutant of pAntp (43–58)-CPG2	409	43848.08	8.95	25.82	91.69	−0.236
**52**	Ala50 substitution mutant of pAntp (43–58)-CPG2	409	43867.13	8.95	26.19	91.69	−0.221
**53**	pAntpHD (Pro50)-CPG2	409	43893.17	8.95	26.66	91.44	−0.229
**54**	Ala51 substitution mutant of pAntp (43–58)-CPG2	409	43881.16	8.95	26.19	91.69	−0.221
**55**	Ala52 substitution mutant of pAntp (43–58)-CPG2	409	43839.07	8.82	24.78	91.69	−0.218
**56**	Met-Arg-CPG2	409	43924.18	8.95	26.00	91.44	−0.233
**57**	Ala54 substitution mutant of pAntp (43–58)-CPG2	409	43864.07	8.95	26.19	91.69	−0.234
**58**	Penetratin-CPG2	409	43924.18	8.95	26.19	91.44	−0.233
**59**	Retro - Tat (57–49)-CPG2	402	43017.05	9.08	30.55	91.09	−0.264
**60**	R6-CPG2	399	42632.57	8.86	32.28	91.78	−0.238
**61**	R9-CPG2	402	43101.13	9.19	36.38	91.09	−0.269
**62**	Crot (27–39) derevative 14-CPG2	402	42971.04	8.53	27.84	91.09	−0.212
**63**	Crot (27–39) derevative 15-CPG2	403	43058.12	8.52	28.27	90.87	−0.214
**64**	Rev (34–50)-CPG2	410	44115.2	9.18	32.92	89.56	−0.309
**65**	HIV-1 Rev (34–50)-CPG2	412	44275.39	9.13	32.60	89.13	−0.302
**66**	Bip6-CPG2	398	42204.11	6.55	25.89	93.97	−0.160
**67**	Bip1-CPG2	398	42264.22	6.55	25.22	93.72	−0.159
**68**	Bip2-CPG2	398	42234.13	6.55	25.41	93.72	−0.166
**69**	Bip16-CPG2	398	42234.09	6.22	26.44	93.72	−0.165
**70**	pAntp (48–58)-CPG2	404	43285.37	8.65	26.33	90.64	−0.229
	CPG2	393	41695.44	6.22	25.33	93.18	−0.173

**Table 5:** Solubility prediction of CPG2 and top 70 CPPs conjugated to both *N*- and C-terminus of CPG2 using the ccsol server.

Peptide Number	Name of the Conjugate	The Solubility Percentage of CPP-CPG2 N-Terminal Conjugates	The Solubility Percentage of CPG2-CPP C-Terminal Conjugates
**1**	Transportan 10 (TP10)-CPG2	86%	86%
**2**	Ala43 substitution mutant of pAntp (43–58)-CPG2	79%	79%
**3**	Crot (27–39) derevative 1-CPG2	87%	84%
**4**	Crot (27–39) derevative 2-CPG2	86%	83%
**5**	Crot (27–39) derevative 3-CPG2	86%	83%
**6**	CyLoP-1-CPG2	85%	82%
**7**	Crot (27–39) derevative 4-CPG2	86%	82%
**8**	Crot (27–39) derevative 5-CPG2	85%	83%
**9**	pAntp (49–58)-CPG2	82%	81%
**10**	Tat (48–60)-CPG2	79%	82%
**11**	pAntp (45–58)-CPG2	80%	79%
**12**	Bip15-CPG2	84%	84%
**13**	pAntp (47–58)-CPG2	81%	79%
**14**	II-CPG2	87%	88%
**15**	Crot (27–39) derevative 6-CPG2	81%	85%
**16**	Crot (27–39) derevative 7-CPG2	84%	85%
**17**	Crot (27–39) derevative 8-CPG2	85%	83%
**18**	Crot (27–39) derevative 9-CPG2	84%	84%
**19**	pAntp (46–58)-CPG2	81%	80%
**20**	7-CPG2	70%	74%
**21**	No.14–2-CPG2	70%	74%
**22**	No.14-CPG2	71%	75%
**23**	No.14–7-CPG2	70%	74%
**24**	Crot (27–39)-CPG2	84%	83%
**25**	Crot (27–39) derevative 10-CPG2	83%	83%
**26**	Crot (27–39) derevative 11-CPG2	83%	83%
**27**	Crot (27–39) derevative 12-CPG2	83%	82%
**28**	pVEC mutant 1-CPG2	80%	80%
**29**	pVEC mutant 2-CPG2	80%	80%
**30**	pVEC mutant 3-CPG2	78%	77%
**31**	Crot (27–39) derevative 13-CPG2	84%	82%
**32**	ARF(1–22)-CPG2	75%	76%
**33**	M918-CPG2	75%	76%
**34**	pAntp (51–58)-CPG2	84%	82%
**35**	pAntp (44–58)-CPG2	79%	79%
**36**	pAntp (50–58)-CPG2	83%	82%
**37**	Ala44 substitution mutant of pAntp (43–58)-CPG2	79%	78%
**38**	PDX -1-PTD-CPG2	79%	78%
**39**	No.14–25-CPG2	73%	75%
**40**	No.14–17-CPG2	72%	73%
**41**	No.14–18-CPG2	71%	72%
**42**	No.14–20-CPG2	72%	73%
**43**	No.14–21-CPG2	72%	73%
**44**	No.14–35-CPG2	70%	72%
**45**	No.14–1-CPG2	71%	72%
**46**	30-CPG2	72%	74%
**47**	Ala45 substitution mutant of pAntp (43–58)-CPG2	79%	78%
**48**	Ala46 substitution mutant of pAntp (43–58)-CPG2	78%	76%
**49**	Ala47 substitution mutant of pAntp (43–58)-CPG2	80%	78%
**50**	Ala48 substitution mutant of pAntp (43–58)-CPG2	81%	80%
**51**	Ala49 substitution mutant of pAntp (43–58)-CPG2	80%	78%
**52**	Ala50 substitution mutant of pAntp (43–58)-CPG2	79%	78%
**53**	pAntpHD (Pro50)-CPG2	79%	78%
**54**	Ala51 substitution mutant of pAntp (43–58)-CPG2	79%	77%
**55**	Ala52 substitution mutant of pAntp (43–58)-CPG2	80%	79%
**56**	Met-Arg-CPG2	79%	78%
**57**	Ala54 substitution mutant of pAntp (43–58)-CPG2	80%	79%
**58**	Penetratin-CPG2	79%	78%
**59**	Retro - Tat (57–49)-CPG2	82%	81%
**60**	R6-CPG2	80%	82%
**61**	R9-CPG2	77%	79%
**62**	Crot (27–39) derevative 14-CPG2	86%	81%
**63**	Crot (27–39) derevative 15-CPG2	85%	81%
**64**	Rev (34–50)-CPG2	73%	74%
**65**	HIV-1 Rev (34–50)-CPG2	74%	73%
**66**	Bip6-CPG2	84%	85%
**67**	Bip1-CPG2	84%	85%
**68**	Bip2-CPG2	84%	85%
**69**	Bip16-CPG2	83%	85%
**70**	pAntp (48–58)-CPG2	82%	79%
	CPG2	83%	83%

**Table 6:** Thermodynamic quantities of CPG2 and 70 top CPPs conjugated to CPG2 using the SCOOP server.

Peptide Number	Name of the Conjugate	*N-Terminal Conjugates*		*C-Terminal Conjugates*	
		*T_m_* (degree~C)	*ΔG_r_* (kcal.mol^−1^)	*T_m_* (degree~C)	*ΔG_r_* (kcal.mol^−1^)
**1**	Transportan 10 (TP10)-CPG2	64.1	−12.3	60.9	−15.9
**2**	Ala43 substitution mutant of pAntp (43-58)-CPG2	59.1	−13.1	60.7	−11.6
**3**	Crot (27−39) derivative 1-CPG2	61.2	−13.3	61.7	−13.1
**4**	Crot (27−39) derivative 2-CPG2	60.7	−14.2	61.7	−13.1
**5**	Crot (27−39) derivative 3-CPG2	61.3	−14.1	59.0	−14.0
**6**	CyLoP-1-CPG2	62.0	−13.2	61.5	−13.6
**7**	Crot (27−39) derivative 4-CPG2	63.1	−14.0	65.8	−13.8
**8**	Crot (27−39) derivative 5-CPG2	62.4	−13.1	60.2	−15.0
**9**	pAntp (49−58)-CPG2	60.5	−16.0	59.3	−16.6
**10**	Tat (48−60)-CPG2	60.1	−13.4	68.0	−13.6
**11**	pAntp (45−58)-CPG2	59.1	−16.1	60.0	−14.1
**12**	Bip15-CPG2	60.7	−12.7	63.1	−14.4
**13**	pAntp (47−58)-CPG2	59.7	−15.4	62.2	−13.2
**14**	II-CPG2	59.7	−15.4	62.5	−14.8
**15**	Crot (27−39) derivative 6-CPG2	62.8	−13.3	62.3	−12.9
**16**	Crot (27−39) derivative 7-CPG2	61.7	−15.9	64.1	−15.2
**17**	Crot (27−39) derivative 8-CPG2	61.1	−14.8	60.3	−15.9
**18**	Crot (27−39) derivative 9-CPG2	59.1	−13.2	61.7	−13.3
**19**	pAntp (46−58)-CPG2	63.0	−12.7	61.2	−14.6
**20**	7-CPG2	62.7	−12.8	60.2	−12.8
**21**	No.14−2-CPG2	61.9	−15.1	59.6	−13.6
**22**	No.14-CPG2	64.7	−15.4	59.8	−14.8
**23**	No.14−7-CPG2	63.6	−14.4	63.0	−12.6
**24**	Crot (27−39)-CPG2	61.4	−15.0	61.0	−14.9
**25**	Crot (27−39) derivative 10-CPG2	60.6	−14.7	57.3	−13.0
**26**	Crot (27−39) derivative 11-CPG2	61.0	−15.1	58.9	−15.4
**27**	Crot (27−39) derivative 12-CPG2	60.5	−13.6	59.5	−13.9
**28**	pVEC mutant 1-CPG2	63.8	−14.0	64.2	−12.0
**29**	pVEC mutant 2-CPG2	60.2	−16.8	57.9	−15.8
**30**	pVEC mutant 3-CPG2	63.8	−11.2	62.1	−13.7
**31**	Crot (27−39) derivative 13-CPG2	64.2	−14.3	58.5	−13.3
**32**	ARF(1−22)-CPG2	61.0	−16.3	65.4	−12.4
**33**	M918-CPG2	65.5	−14.8	63.3	−12.6
**34**	pAntp (51−58)-CPG2	56.9	−15.8	61.6	−15.3
**35**	pAntp (44−58)-CPG2	58.1	−13.6	59.5	−16.8
**36**	pAntp (50−58)-CPG2	62.7	−15.6	61.0	−13.6
**37**	Ala44 substitution mutant of pAntp (43−58)-CPG2	60.7	−13.8	58.4	−15.6
**38**	PDX -1-PTD-CPG2	57.6	−13.9	58.5	−14.2
**39**	No.14−25-CPG2	62.3	−11.5	58.5	−15.3
**40**	No.14−17-CPG2	60.3	−14.6	58.7	−14.9
**41**	No.14−18-CPG2	60.6	−13.3	61.6	−13.9
**42**	No.14−20-CPG2	62.3	−14.1	56.6	−15.2
**43**	No.14−21-CPG2	63.8	−14.8	60.8	−14.2
**44**	No.14−35-CPG2	62.5	−15.9	59.9	−15.4
**45**	No.14−1-CPG2	61.7	−15.5	60.7	−13.4
**46**	30-CPG2	62.8	−13.8	58.3	−15.0
**47**	Ala45 substitution mutant of pAntp (43−58)-CPG2	60.2	−14.0	59.7	−16.3
**48**	Ala46 substitution mutant of pAntp (43−58)-CPG2	63.2	−12.9	60.4	−14.6
**49**	Ala47 substitution mutant of pAntp (43−58)-CPG2	60.6	−13.6	61.3	−13.0
**50**	Ala48 substitution mutant of pAntp (43−58)-CPG2	62.2	−14.0	57.1	−16.1
**51**	Ala49 substitution mutant of pAntp (43−58)-CPG2	61.1	−14.6	60.7	−14.7
**52**	Ala50 substitution mutant of pAntp (43−58)-CPG2	60.8	−13.3	59.5	−15.3
**53**	pAntpHD (Pro50)-CPG2	62.7	−12.7	60.7	−13.4
**54**	Ala51 substitution mutant of pAntp (43−58)-CPG	60.3	−14.9	56.6	−13.4
**55**	Ala52 substitution mutant of pAntp (43−58)-CPG2	63.9	−11.9	61.1	−13.4
**56**	Met-Arg-CPG2	64.2	−14.3	59.6	−14.3
**57**	Ala54 substitution mutant of pAntp (43−58)-CPG2	60.4	−14.7	59.5	−14.9
**58**	Penetratin-CPG2	60.4	−15.1	60.0	−12.5
**59**	Retro - Tat (57−49)-CPG2	61.0	−14.0	60.4	−14.7
**60**	R6-CPG2	58.7	−14.3	62.0	−14.4
**61**	R9-CPG2	62.7	−13.8	57.7	−15.5
**62**	Crot (27−39) derivative 14-CPG2	63.5	−13.2	62.6	−13.0
**63**	Crot (27−39) derivative 15-CPG2	60.3	−13.6	63.0	−15.1
**64**	Rev (34−50)-CPG2	59.1	−16.4	58.5	−16.0
**65**	HIV-1 Rev (34−50)-CPG2	62.0	−13.3	59.9	−15.2
**66**	Bip6-CPG2	62.6	−15.0	62.6	−13.9
**67**	Bip1-CPG2	56.7	−14.9	63.4	−11.2
**68**	Bip2-CPG2	60.8	−13.7	60.5	−13.0
**69**	Bip16-CPG2	61.8	−14.6	61.4	−14.3
**70**	pAntp (48−58)-CPG2	56.5	−15.6	59.5	−13.5
	CPG2	61.7	−15.1	61.7	−15.1

**Table 7:** Result of the stability analyses of CPG2 and top 70 CPPs conjugated to CPG2 using the FoldX suite server.

Peptide Number	Name of the Conjugate	*N-Terminal Conjugates*		*C-Terminal Conjugates*	
		*Δ*G _side H bond_ (kcal.mol^−1^)	*Δ*G_total_ (kcal.mol^−1^)	*Δ*G _side H bond_ (kcal.mol^−1^)	*Δ*G_total_ (kcal.mol^−1^)
**1**	Transportan 10 (TP10)-CPG2	−129.40	225.54	−109.75	280.94
**2**	Ala43 substitution mutant of pAntp (43-58)-CPG2	−122.58	276.34	−116.80	290.77
**3**	Crot (27–39) derivative 1-CPG2	−124.28	212.60	−116.83	231.74
**4**	Crot (27–39) derivative 2-CPG2	−118.09	244.42	−116.83	231.74
**5**	Crot (27–39) derivative 3-CPG2	−124.84	201.96	−116.51	221.44
**6**	CyLoP-1-CPG2	−114.78	222.25	−121.71	237.72
**7**	Crot (27–39) derivative 4-CPG2	−118.05	240.24	−117.87	258.05
**8**	Crot (27–39) derivative 5-CPG2	−111.72	242.79	−122.19	226.79
**9**	pAntp (49–58)-CPG2	−117.91	213.28	−128.23	192.93
**10**	Tat (48–60)-CPG2	−113.01	237.15	−110.09	285.79
**11**	pAntp (45–58)-CPG2	−109.21	257.80	−123.56	222.57
**12**	Bip15-CPG2	−111.99	219.08	−110.39	206.12
**13**	pAntp (47–58)-CPG2	−115.32	251.89	−119.12	257.36
**14**	II-CPG2	−118.36	220.02	−120.90	269.15
**15**	Crot (27–39) derivative 6-CPG2	−122.01	240.34	−112.90	247.76
**16**	Crot (27–39) derivative 7-CPG2	−114.80	218.64	−104.19	264.74
**17**	Crot (27–39) derivative 8-CPG2	−113.66	231.91	−118.05	228.78
**18**	Crot (27–39) derivative 9-CPG2	−129.44	213.50	−116.83	261.88
**19**	pAntp (46–58)-CPG2	−129.66	234.61	−122.16	220.00
**20**	7-CPG2	−118.37	248.66	−113.78	227.61
**21**	No.14–2-CPG2	−128.25	266.61	−106.97	305.73
**22**	No.14-CPG2	−121.58	288.14	−125.08	244.91
**23**	No.14–7-CPG2	−106.30	268.12	−120.60	253.67
**24**	Crot (27–39)-CPG2	−121.32	239.73	−121.87	211.89
**25**	Crot (27–39) derivative 10-CPG2	−123.15	236.04	−119.18	232.36
**26**	Crot (27–39) derivative 11-CPG2	−117.73	240.33	−120.16	223.44
**27**	Crot (27–39) derivative 12-CPG2	−119.24	236.96	−125.03	239.37
**28**	pVEC mutant 1-CPG2	−122.35	279.77	−112.86	279.81
**29**	pVEC mutant 2-CPG2	−118.54	275.22	−114.38	236.84
**30**	pVEC mutant 3-CPG2	−123.99	238.03	−118.77	231.76
**31**	Crot (27–39) derivative 13-CPG2	−117.23	274.19	−124.87	208.41
**32**	ARF(1–22)-CPG2	−116.67	287.55	−127.44	277.30
**33**	M918-CPG2	−100.79	353.34	−120.74	304.20
**34**	pAntp (51–58)-CPG2	−120.11	213.01	−119.48	227.21
**35**	pAntp (44–58)-CPG2	−120.79	271.81	−113.18	237.39
**36**	pAntp (50–58)-CPG2	−117.81	219.53	−116.73	245.44
**37**	Ala44 substitution mutant of pAntp (43-58)-CPG2	−131.75	260.06	−107.05	248.14
**38**	PDX -1-PTD-CPG2	−118.12	230.27	−123.45	254.56
**39**	No.14–25-CPG2	−117.33	259.77	−111.95	223.28
**40**	No.14–17-CPG2	−122.74	201.85	−124.49	287.89
**41**	No.14–18-CPG2	−110.20	279.65	−111.53	235.99
**42**	No.14–20-CPG2	−113.46	286.37	−120.35	235.61
**43**	No.14–21-CPG2	−123.65	306.20	−103.28	271.59
**44**	No.14–35-CPG2	−118.98	316.52	−127.82	288.57
**45**	No.14–1-CPG2	−117.56	288.64	−111.92	232.73
**46**	30-CPG2	−114.00	274.45	−112.39	225.22
**47**	Ala45 substitution mutant of pAntp (43–58)-CPG2	−121.63	244.81	−128.84	256.18
**48**	Ala46 substitution mutant of pAntp (43–58)-CPG2	−114.00	258.27	−109.97	275.43
**49**	Ala47 substitution mutant of pAntp (43–58)-CPG2	−111.21	279.55	−118.80	250.65
**50**	Ala48 substitution mutant of pAntp (43–58)-CPG2	−115.90	215.33	−113.15	225.09
**51**	Ala49 substitution mutant of pAntp (43–58)-CPG2	−120.58	259.79	−125.14	244.61
**52**	Ala50 substitution mutant of pAntp (43–58)-CPG2	−122.21	266.42	−117.50	275.80
**53**	pAntpHD (Pro50)-CPG2	−116.73	247.08	−113.98	279.80
**54**	Ala51 substitution mutant of pAntp (43–58)-CPG2	−120.61	256.74	−117.29	277.38
**55**	Ala52 substitution mutant of pAntp (43–58)-CPG2	−114.28	273.55	−127.58	224.55
**56**	Met-Arg-CPG2	−120.88	294.89	−130.43	225.80
**57**	Ala54 substitution mutant of pAntp (43–58)-CPG2	−113.18	274.17	−126.16	223.46
**58**	Penetratin-CPG2	−114.09	237.56	−114.95	232.56
**59**	Retro - Tat (57–49)-CPG2	−104.71	239.28	−115.58	209.58
**60**	R6-CPG2	−108.43	215.13	−120.23	219.49
**61**	R9-CPG2	−126.43	207.16	−138.39	218.37
**62**	Crot (27–39) derivative 14-CPG2	−116.15	187.59	−117.38	233.60
**63**	Crot (27–39) derivative 15-CPG2	−109.48	259.98	−112.67	226.84
**64**	Rev (34–50)-CPG2	−140.73	250.30	−120.99	267.37
**65**	HIV-1 Rev (34–50)-CPG2	−123.81	241.35	−120.42	237.54
**66**	Bip6-CPG2	−126.11	176.25	−117.74	237.91
**67**	Bip1-CPG2	−120.74	163.93	−125.52	199.54
**68**	Bip2-CPG2	−106.63	227.56	−122.97	213.03
**69**	Bip16-CPG2	−118.69	199.30	−126.85	187.15
**70**	pAntp (48–58)-CPG2	−121.70	239.55	−125.92	230.92
	CPG2	−124.91	203.85	−124.91	203.85

**Table 8:** Result of folding rate prediction using the Foldrate server and backbone flexibility analysis by the Dynamine server for CPG2 and 70 top CPPs conjugated to CPG2.

Peptide Number	Name of the Conjugate	*N-Terminal Conjugates*	*C-Terminal Conjugates*
		Predicted Half-Folding Time (sec)	Predicted Half-Folding Time (sec)
**1**	Transportan 10 (TP10)-CPG2	315.75	292.07
**2**	Ala43 substitution mutant of pAntp (43-58)-CPG2	341.86	375.47
**3**	Crot (27–39) derivative 1-CPG2	269.35	294.03
**4**	Crot (27–39) derivative 2-CPG2	266.25	297.08
**5**	Crot (27–39) derivative 3-CPG2	266.25	297.08
**6**	CyLoP-1-CPG2	263.17	296.95
**7**	Crot (27–39) derivative 4-CPG2	290.06	299.60
**8**	Crot (27–39) derivative 5-CPG2	251.58	269.07
**9**	pAntp (49–58)-CPG2	260.00	265.84
**10**	Tat (48–60)-CPG2	250.01	261.29
**11**	pAntp (45–58)-CPG2	326.14	336.71
**12**	Bip15-CPG2	254.99	251.17
**13**	pAntp (47–58)-CPG2	312.02	325.60
**14**	II-CPG2	282.10	260.72
**15**	Crot (27–39) derivative 6-CPG2	272.11	300.16
**16**	Crot (27–39) derivative 7-CPG2	300.50	310.30
**17**	Crot (27–39) derivative 8-CPG2	262.57	286.80
**18**	Crot (27–39) derivative 9-CPG2	251.02	271.45
**19**	pAntp (46–58)-CPG2	314.64	324.84
**20**	7-CPG2	304.54	328.42
**21**	No.14–2-CPG2	303.00	306.34
**22**	No.14-CPG2	312.62	340.53
**23**	No.14–7-CPG2	288.59	325.49
**24**	Crot (27–39)-CPG2	262.67	283.81
**25**	Crot (27–39) derivative 10-CPG2	263.85	297.45
**26**	Crot (27–39) derivative 11-CPG2	287.51	303.33
**27**	Crot (27–39) derivative 12-CPG2	281.35	296.93
**28**	pVEC mutant 1-CPG2	318.66	318.66
**29**	pVEC mutant 2-CPG2	308.37	318.66
**30**	pVEC mutant 3-CPG2	331.94	310.97
**31**	Crot (27–39) derivative 13-CPG2	257.41	310.97
**32**	ARF(1–22)-CPG2	375.57	426.04
**33**	M918-CPG2	371.56	371.56
**34**	pAntp (51–58)-CPG2	246.89	371.56
**35**	pAntp (44–58)-CPG2	341.31	359.69
**36**	pAntp (50–58)-CPG2	253.19	371.56
**37**	Ala44 substitution mutant of pAntp (43–58)-CPG2	339.30	371.56
**38**	PDX -1-PTD-CPG2	354.49	371.56
**39**	No.14–25-CPG2	302.06	325.92
**40**	No.14–17-CPG2	286.95	325.92
**41**	No.14–18-CPG2	316.39	325.92
**42**	No.14–20-CPG2	300.29	325.92
**43**	No.14–21-CPG2	303.60	341.55
**44**	No.14–35-CPG2	309.58	341.55
**45**	No.14–1-CPG2	315.26	341.55
**46**	30-CPG2	311.23	328.53
**47**	Ala45 substitution mutant of pAntp (43–58)-CPG2	321.46	368.26
**48**	Ala46 substitution mutant of pAntp (43–58)-CPG2	341.11	368.26
**49**	Ala47 substitution mutant of pAntp (43–58)-CPG2	301.29	364.51
**50**	Ala48 substitution mutant of pAntp (43–58)-CPG2	301.06	364.51
**51**	Ala49 substitution mutant of pAntp (43–58)-CPG2	328.09	338.69
**52**	Ala50 substitution mutant of pAntp (43–58)-CPG2	342.91	357.57
**53**	pAntpHD (Pro50)-CPG2	324.61	342.27
**54**	Ala51 substitution mutant of pAntp (43–58)-CPG2	342.47	342.27
**55**	Ala52 substitution mutant of pAntp (43–58)-CPG2	300.29	364.03
**56**	Met-Arg-CPG2	298.31	315.14
**57**	Ala54 substitution mutant of pAntp (43–58)-CPG2	295.42	305.35
**58**	Penetratin-CPG2	354.39	400.84
**59**	Retro - Tat (57–49)-CPG2	243.94	246.69
**60**	R6-CPG2	255.43	249.84
**61**	R9-CPG2	269.07	249.03
**62**	Crot (27–39) derivative 14-CPG2	258.50	276.26
**63**	Crot (27–39) derivative 15-CPG2	255.15	263.85
**64**	Rev (34–50)-CPG2	265.86	251.26
**65**	HIV-1 Rev (34–50)-CPG2	276.22	261.16
**66**	Bip6-CPG2	239.77	256.43
**67**	Bip1-CPG2	256.15	259.01
**68**	Bip2-CPG2	240.98	260.66
**69**	Bip16-CPG2	242.18	262.04
**70**	pAntp (48–58)-CPG2	300.95	278.92
	CPG2	229.89	229.89

**Table 9:** Analysis of top 70 CPPs and top 70 CPPs conjugated to CPG2 for in vivo application.

Peptide Number	CPPs’ Name	Immunogenicity of Respective CPP-CPG2 /CPG2-CPP Conjugate		Toxicity of CPPs	Hemolysis Potency of CPPs (PROB Score)
		N-Terminal Conjugates	C-Terminal Conjugates		
**1**	Transportan 10 (TP10)	0.6620	0.6790	non-toxin	0.83
**2**	Ala43 substitution mutant of pAntp (43–58)	0.6874	0.6907	non-toxin	0.48
**3**	Crot (27–39) derivative 1	0.7201	0.7267	Toxin	0.49
**4**	Crot (27–39) derivative 2	0.7199	0.7263	Toxin	0.49
**5**	Crot (27–39) derivative 3	0.7238	0.7319	Toxin	0.49
**6**	CyLoP-1	0.7244	0.7323	Toxin	0.49
**7**	Crot (27–39) derivative 4	0.7361	0.7403	non-toxin	0.49
**8**	Crot (27–39) derivative 5	0.7276	0.7334	Toxin	0.49
**9**	pAntp (49–58)	0.6973	0.7017	non-toxin	0.48
**10**	Tat (48–60)	0.7208	0.7385	non-toxin	0.49
**11**	pAntp (45–58)	0.6910	0.6794	non-toxin	0.49
**12**	Bip15	0.6929	0.6903	non-toxin	0.49
**13**	pAntp (47–58)	0.6855	0.6690	non-toxin	0.48
**14**	II	0.6466	0.6521	non-toxin	0.83
**15**	Crot (27–39) derivative 6	0.7219	0.7300	Toxin	0.49
**16**	Crot (27–39) derivative 7	0.7439	0.7410	non-toxin	0.49
**17**	Crot (27–39) derivative 8	0.7267	0.7314	Toxin	0.49
**18**	Crot (27–39) derivative 9	0.7263	0.7363	Toxin	0.49
**19**	pAntp (46–58)	0.6813	0.6764	non-toxin	0.49
**20**	7	0.6942	0.6961	non-toxin	0.49
**21**	No.14–2	0.6961	0.6980	non-toxin	0.49
**22**	No.14	0.6915	0.6934	non-toxin	0.49
**23**	No.14–7	0.6844	0.6863	non-toxin	0.49
**24**	Crot (27–39)	0.7371	0.7409	Toxin	0.49
**25**	Crot (27–39) derivative 10	0.7366	0.7388	Toxin	0.49
**26**	Crot (27–39) derivative 11	0.7344	0.7406	Toxin	0.49
**27**	Crot (27–39) derivative 12	0.7307	0.7327	non-toxin	0.49
**28**	pVEC mutant 1	0.7006	0.6886	non-toxin	0.32
**29**	pVEC mutant 2	0.6874	0.6755	non-toxin	0.32
**30**	pVEC mutant 3	0.6901	0.6782	non-toxin	0.31
**31**	Crot (27–39) derivative 13	0.7304	0.7344	Toxin	0.49
**32**	ARF(1–22)	0.6848	0.6917	non-toxin	0.48
**33**	M918	0.6865	0.6893	non-toxin	0.48
**34**	pAntp (51–58)	0.7023	0.7142	non-toxin	0.49
**35**	pAntp (44–58)	0.6892	0.6913	non-toxin	0.49
**36**	pAntp (50–58)	0.7037	0.7153	non-toxin	0.49
**37**	Ala44 substitution mutant of pAntp (43–58)	0.6840	0.6866	non-toxin	0.48
**38**	PDX -1-PTD	0.6845	0.6861	non-toxin	0.49
**39**	No.14–25	0.6910	0.6907	non-toxin	0.49
**40**	No.14–17	0.6889	0.6900	non-toxin	0.49
**41**	No.14–18	0.6927	0.6938	non-toxin	0.49
**42**	No.14–20	0.6922	0.6933	non-toxin	0.48
**43**	No.14–21	0.6949	0.6961	non-toxin	0.48
**44**	No.14–35	0.6924	0.6938	non-toxin	0.49
**45**	No.14–1	0.6929	0.6941	non-toxin	0.49
**46**	30	0.6981	0.7012	non-toxin	0.49
**47**	Ala45 substitution mutant of pAntp (43–58)	0.6741	0.6817	non-toxin	0.47
**48**	Ala46 substitution mutant of pAntp (43–58)	0.6756	0.6765	non-toxin	0.47
**49**	Ala47 substitution mutant of pAntp (43–58)	0.6740	0.6779	non-toxin	0.47
**50**	Ala48 substitution mutant of pAntp (43–58)	0.7007	0.7046	non-toxin	0.48
**51**	Ala49 substitution mutant of pAntp (43–58)	0.6784	0.6823	non-toxin	0.48
**52**	Ala50 substitution mutant of pAntp (43–58)	0.6827	0.6865	non-toxin	0.48
**53**	pAntpHD (Pro50)	0.6887	0.6926	non-toxin	0.49
**54**	Ala51 substitution mutant of pAntp (43–58)	0.6878	0.6916	non-toxin	0.48
**55**	Ala52 substitution mutant of pAntp (43–58)	0.6932	0.6970	non-toxin	0.48
**56**	Met-Arg	0.6792	0.6833	non-toxin	0.48
**57**	Ala54 substitution mutant of pAntp (43–58)	0.6791	0.6828	non-toxin	0.48
**58**	Penetratin	0.6820	0.6859	non-toxin	0.48
**59**	Retro - Tat (57–49)	0.7094	0.7274	non-toxin	0.17
**60**	R6	0.6983	0.7184	non-toxin	0.49
**61**	R9	0.7084	0.7284	non-toxin	0.49
**62**	Crot (27–39) derivative 14	0.7263	0.7208	Toxin	0.49
**63**	Crot (27–39) derivative 15	0.7182	0.7284	Toxin	0.49
**64**	Rev (34–50)	0.7032	0.7193	non-toxin	0.49
**65**	HIV-1 Rev (34–50)	0.7128	0.7213	non-toxin	0.48
**66**	Bip6	0.6956	0.6980	non-toxin	0.49
**67**	Bip1	0.6920	0.6924	non-toxin	0.49
**68**	Bip2	0.6945	0.6984	non-toxin	0.49
**69**	Bip16	0.6957	0.6968	non-toxin	0.49
**70**	pAntp (48–58)	0.6838	0.6695	non-toxin	0.47
	CPG2	0.7012	0.7012	-	-

## Supplementary Materials

The followings are available online: Table S1: Result of CPPred-RF server analysis for all 1155 CPPs, Table S2: Result of analysis with mfold server for mRNA intiation translation region sequences of CPG2 and top 70 CPPs conjugated to *N*-terminal of CPG2 in PET 14b expression vector (All C-terminal conjugates have the same result as that of CPG2 itself), Table S3: Physiochemical properties of top 70 CPPs analyzed via ProtParam tool, Table S4: Physiochemical properties of top 70 CPG2-CPP C-terminal conjugates analyzed via ProtParam tool, Table S5: Analysis of 3D modeled structures of CPG2 and top 70 CPPs conjugated to CPG2 by PDBsum server, Table S6: Thermodynamic properties CPG2 and CPP-CPG2 conjugates calculated by the SCOOP sever, Table S7: Thermodynamic energies of CPG2 and CPP-CPG2 conjugates calculated by FoldX, Table S8: Analysis of aggregation possibility for CPG2 and top 70 CPPs conjugated to CPG2 by Pasta 2.0 and AGGRESCAN servers, Table S9: Half-life prediction for top 70 CPPs conjugated with CPG2, Table S10: Selection of top CPP candidates in conjugation to CPG2, Table S11: Shows whether the conjugates or CPG2 will be cleaved by respective proteases or not. The probability score of 0.700 was considered as significant, Table S12: Shows whether the conjugates will have increased risk of cleavage by each protease due to CPP addition compared with CPG2. Probability score of 0.700 was considered significant.
